# Active steering of cathodoluminescence through a generalized Smith–Purcell effect

**DOI:** 10.1038/s41377-026-02280-y

**Published:** 2026-05-06

**Authors:** Eduardo J. C. Dias, A. Rodríguez Echarri, Theis P. Rasmussen, F. Javier García de Abajo, Joel D. Cox

**Affiliations:** 1 POLIMA—Center for Polariton-driven Light–Matter Interactions, University of Southern Denmark, Odense M, Denmark; 2 Center for Nanophotonics, NWO Institute AMOLF, Amsterdam, The Netherlands; 3 Max-Born-Institut, Berlin, Germany; 4 ICFO-Institut de Ciencies Fotoniques, The Barcelona Institute of Science and Technology, Barcelona, Spain; 5 ICREA-Institució Catalana de Recerca i Estudis Avançats, Barcelona, Spain; 6 Danish Institute for Advanced Study, University of Southern Denmark, Odense M, Denmark

**Keywords:** Metamaterials, Optoelectronic devices and components, Photonic devices, Nanophotonics and plasmonics

## Abstract

Optical metasurfaces can shape the near fields of energetic electrons, enabling Smith–Purcell (SP) emission. We introduce a generalized SP effect relying on finite periodic arrays whose elements possess individually tunable polarizabilities, allowing us to explore higher-order SP radiation. By controlling the amplitude and phase of each of the elements, we show through rigorous theory the ability to create an SP steering device. In particular, we explore the active tuning capabilities of doped graphene, and thermally driven phase-change materials, which we compare with standard passive plasmonic structures made of gold and silver. Our results establish programmable electron-driven light sources and spectroscopic probes spanning the terahertz-to-visible range, advancing tunable metasurfaces for next-generation electron-photon technologies.

## Introduction

Optical metasurfaces have recently emerged as two-dimensional (2D) platforms for developing compact devices capable of manipulating light at the nanoscale^1,2^. The functionalities of metasurfaces are rich and diverse, including nonlinear frequency conversion^3–5^, optical holography^6,7^, and wavefront shaping^8^, just to mention a few. The foundation of these various applications builds upon periodic arrangements of subwavelength nanostructures, engineered such that the localized excitation of all the individual constituents converges to shape an overall targeted scattering response. Furthermore, the combination of the 2D metasurfaces—being easier to fabricate than their 3D metamaterial counterparts—along with a typically lower degree of losses, endows these planar architectures with unique advantages for many applications in nanophotonics.

While metasurfaces are conventionally used to manipulate light impinging from the far-field^9^, the control of optical near-fields by metasurfaces has been demonstrated both theoretically and experimentally^10,11^. In this context, energetic free electrons serve as an excellent source of broadband evanescent electromagnetic fields, which can be directed over a metasurface with exceptional spatial precision^12^. Specifically, the optical excitation of elements in a structured surface by swift electrons can generate light emission covering a vast range of frequencies, thus holding significant promise for applications using light sources at the nanoscale^13–15^.

In particular, free-electron-induced Smith–Purcell (SP) radiation, in which the time-varying Coulomb field of a charged particle moving parallel to a periodically structured surface generates directional radiation through constructive interference of cathodoluminescence (CL), has been studied extensively since its original experimental demonstration^16^, with subsequent experiments establishing its broadband nature, angular dispersion, and robustness across electron energies^17–20^. Building on these foundations, engineered structures have been used to tailor SP emission. These include periodic arrays of metallic or dielectric scatterers^21,22^, metasurface and metagrating geometries for controlling directionality and angular dispersion^23–25^, chirped or aperiodic gratings that redistribute emission across multiple channels^26^, and Babinet-type complementary designs enabling polarization and symmetry control^27^. SP radiation has also been explored in polaritonic platforms, where the coupling between free electrons, lattice resonances, and surface plasmon polaritons leads to modified dispersion relations and rich angle–frequency emission landscapes^12,28,29^. Beyond tailoring out-coupled SP light, recent work has shown that the same momentum-matching mechanism can be used to directly excite surface polaritons, enabling in-plane SP excitation of plasmonic and phonon-polaritonic waves^30–32^.

SP radiation depends crucially on both the geometrical and intrinsic material properties of a metasurface. While the geometry of metal gratings supporting collective free electron excitations is typically controlled passively (e.g., by changing the size of individual elements and their periodicity), intrinsic material parameters, such as dielectric permittivity, are rather challenging to control. Further customization of SP emission can be obtained by relaxing stringent conditions on the periodicity of an array, with aperiodic structures providing more complex far-field emission patterns^26^ and near-field focusing^23^. However, most systems explored in this context lack the ability to actively control the emission properties of SP radiation, such as directionality, far-field amplitude, and polarization.

In this work, we present a generalized study of SP radiation in finite-size periodic arrays with individually tunable polarizable elements. Opening with a summary of conventional SP radiation in periodic arrays of identical scatterers, we generalize the formalism to investigate the far-field emission characteristics of finite arrays of arbitrary dipoles, emphasizing the coupling to discrete emission channels with specific directionality. The generalized SP formalism is then applied to steer the CL produced by an electron passing over a periodic array of non-uniform elements by customizing the polarizability of each scatterer. We explore this concept by simulating generalized SP emission in paradigmatic actively tunable nanophotonic architectures: a periodic graphene nanoribbon array, supporting electrically tunable plasmon resonances, and particles comprised of phase-change materials that can be optically activated through variations in temperature. Our results open new avenues for electron spectroscopy, light sources, photon-electron interactions, and optimizing the inverse SP effect used in laser-driven linear accelerators.

## Results

### Generalized SP condition

**Fig. 1:**
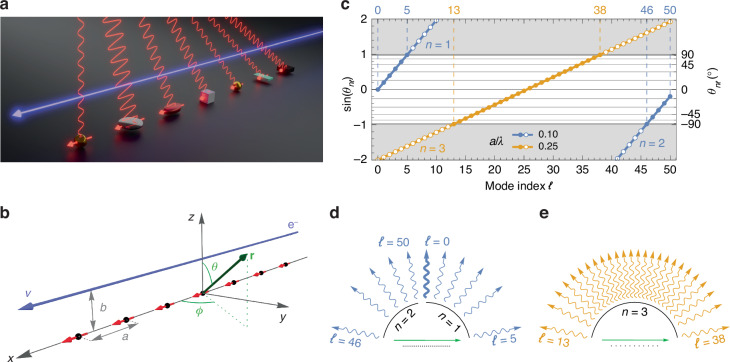
Generalized SP emission. **a** Artistic sketch of the considered system, composed of a periodic array of scatterers interacting with an electron beam moving parallel to the array. **b** Simplified scheme of the sketch in (**a**), where we indicate the period *a* of the array, the electron (e^−^) velocity *v*, and impact parameter *b*. We further indicate the adopted coordinate system and the polar and azimuthal angles *θ* and *ϕ* for a general position vector **r**. All scatterers are taken as point dipoles with dipole moments indicated by red arrows. **c** Generalized Smith–Purcell (GSP) emission condition in the *ϕ* = 0 plane, given by Eq. (6), for an electron with velocity *v* = 0.1 *c* passing near an array with *N* = 51 elements and period indicated by the legend. The SP order *n* for each curve is marked next to it, while all remaining values of *n* lie outside the plot region for the considered values of *a*/*λ*. The gray areas indicate the regions where $| \sin {\theta }_{n\ell }| > 1$ and therefore constructive interference is kinematically forbidden. Dots inside (outside) this region are marked as unfilled (filled), and the corresponding emission angle *θ*_*nℓ*_, when allowed, is denoted along the right axis. **d**, **e** Schematic illustrations of the available GSP emission channels for each of the color-coordinated arrays in (**c**). Each arrow corresponds to a specific *n*, *ℓ* pair (see labels) and is represented outgoing at the *θ*_*nℓ*_ angle. **d** The *ℓ* = 0 case is highlighted by a thicker arrow, denoting the conventional SP emission of a uniform array

We start by considering a general one-dimensional array composed of *N* elements periodically placed at coordinates ${{\bf{r}}}_{j}=ja\hat{{\bf{x}}}$ (*j* = 0, ⋯ , *N* − 1) along the *x* axis, where *a* is the lattice period, as depicted in Fig. 1a, b. The array is surrounded by vacuum and is excited by a swift electron passing at a distance *b* > 0 and moving with velocity ${\bf{v}}=v\hat{{\bf{x}}}$ that generates at frequency *ω* (i.e., wavelength *λ* = 2*πc*/*ω*) an external field **E**^ext^(**r**_*j*_) given by Eq. (9) in “Methods” section. In general, we assume that each element *j* in the array can exhibit a distinct polarizability response *α*_*j*_, and thus will generate a distinct dipole moment

1 $${{\bf{p}}}_{j}={\alpha }_{j}\cdot \left[{{\bf{E}}}^{\mathrm{ext}}({{\bf{r}}}_{j})+\mathop{\sum }\limits_{i\ne j}{{\mathcal{G}}}_{ji}\cdot {{\bf{p}}}_{i}\right]$$

where *k* = *ω*/*c* = 2*π*/*λ* is the free space optical wave vector and the term ${{\mathcal{G}}}_{ji}=({k}^{2}+\nabla \otimes \nabla ){\rm e}^{{\rm i}k{r}_{ji}}/{r}_{ji}$ represents the dipole-dipole interaction between array elements *i* and *j* in terms of their distance *r*_*ji*_ = ∣**r**_*j*_ − **r**_*i*_∣. The self-consistent equation above can be inverted to find the induced dipole moment in each array element, which we can write in the form ${{\bf{p}}}_{j}={{\bf{p}}}_{j}^{0}{\rm e}^{{\rm i}\omega ja/v}$, anticipating from Eq. (9) that the time delay associated with the finite electron velocity introduces a phase difference of *ωa*/*v* between two consecutive dipole elements. In general, each dipole ${{\bf{p}}}_{j}^{0}$ may contain components along *x* and *z*, but components along *y* are forbidden by symmetry.

The far-field CL emission by such an array, as a function of emission direction $\hat{{\bf{r}}}=(\sin \theta \cos \phi ,\sin \theta \sin \phi ,\cos \theta )$, i.e., along polar and azimuthal angles *θ* and *ϕ*, respectively, represented in Fig. 1b, can be constructed by the superposition of the far-field emitted by each array element^33^,

2 $${\bf{f}}(\hat{{\bf{r}}})={k}^{2}(1-\hat{{\bf{r}}}\otimes \hat{{\bf{r}}})\cdot \mathop{\sum }\limits_{j}{{\bf{p}}}_{j}^{0}{\rm e}^{{\rm i}kja/\beta }{\rm e}^{-{\rm i}k({{\bf{r}}}_{j}\cdot \hat{{\bf{r}}})}$$

where *β* = *v*/*c* and we introduce a phase $k({{\bf{r}}}_{j}\cdot \hat{{\bf{r}}})=kaj\sin \theta \cos \phi$ to account for the optical path difference between the field emitted by two consecutive array elements. In Eq. (2), the leading factor in parentheses corresponds to an angular envelope determined by the orientation of each dipole, and the *j*-sum encodes the far-field interference pattern arising from all radiating elements.

In traditional SP emission, an infinite array of identical elements is considered, for which we find ${{\bf{p}}}_{j}^{0}={{\bf{p}}}^{0}$ for all values of *j*. Under such conditions, the far-field amplitude is nonvanishing only if the combined phases in Eq. (2) sum to an integer multiple of 2*π*, which, at the *xz* plane defined by *ϕ* = 0 (or 2*π*), leads to the usual SP condition,

3 $$\sin {\theta }_{n}=\frac{1}{\beta }-\frac{n\lambda }{a}$$

where $n\in {\mathbb{Z}}$ denotes the SP order (at planes where *ϕ* ≠ 0, the right-hand side of Eq. (3) needs to be modified by a global $1/\cos \phi$ factor that shifts the SP emission angle *θ*_*n*_ provided that $| \sin {\theta }_{n}|$ remains smaller than 1). Along directions that satisfy the condition above, all array elements radiate in phase, producing a far-field lobe that is locally a plane wave at its peak and whose angular width decreases with increasing *N*. Importantly, the array interference does not introduce any polarization mechanisms, so the polarization within each lobe is set by the radiation pattern of the individual emitters (i.e., by the orientation of the induced dipoles). If dipoles with different orientations are present, this applies component-by-component, so the polarization observed in a given lobe follows from the coherent superposition of the corresponding vector components.

Equation (3) holds only when the induced dipole is uniform across all array elements, which is satisfied if the array is infinite and all its elements are equivalent, but breaks down if one of those conditions is not met. Instead, in the general case where the dipoles respond non-uniformly to the external field (and we are no longer able to remove the terms ${{\bf{p}}}_{j}^{0}$ from the *j*-sum), it is convenient to introduce the Fourier decomposition of the induced dipole array, ${{\bf{p}}}_{j}^{0}={\sum }_{\ell }{\widetilde{{\bf{p}}}}_{\ell }{\rm e}^{2\pi {\rm i}j\ell /N}$, which allows us to rewrite the sum in Eq. (2) as

4 $$\mathop{\sum }\limits_{j}{{\bf{p}}}_{j}^{0}{\rm e}^{{\rm i}kja/\beta }{\rm e}^{-{\rm i}kja(\hat{{\bf{r}}}\cdot \hat{{\bf{x}}})}=\mathop{\sum }\limits_{\ell }{\widetilde{{\bf{p}}}}_{\ell }\mathop{\sum }\limits_{j}{\rm e}^{{\rm i}j{\chi }_{\ell }}$$

with ${\chi }_{\ell }=2\pi \ell /N+ka(1/\beta -\hat{{\bf{r}}}\cdot \hat{{\bf{x}}})$. By doing so, we are able to decouple the dipole moment (now written in terms of the harmonic array modes denoted by *ℓ*) from the array positions (denoted by *j*), which, crucially, allows us to analytically evaluate the *j*-sum in Eq. (4) as

5 $$\mathop{\sum }\limits_{j}{{\mathrm{e}}}^{{\mathrm{i}}j{\chi }_{\ell }}=\left\{\begin{array}{l}\begin{array}{cc}{{\mathrm{e}}}^{{\mathrm{i}}(N-1){\chi }_{\ell }/2}\left[\frac{\sin (N{\chi }_{\ell }/2)}{\sin ({\chi }_{\ell }/2)}\right], & {\chi }_{\ell}\ne 2\pi n\end{array}\\ \begin{array}{cc}N,\,\,\,\,\,\,\,\,\,\,\,\,\,\,\,\,\,\,\,\,\,\,\,\,\,\,\,\,\,\,\,\,\,\,\,\,\,\,\,\,\,\,\,\,\,\,\,\,\,\,\, & {\chi }_{\ell }=2\pi n\end{array}\end{array}\right.$$

where, as above, we take *n* to be an integer number. In this case, the far-field angular distribution can be readily found by Eqs. (2), (4), and (5), and exhibits plane-wave-like emission lobes at resonances signaled by the condition *χ*_*ℓ*_ = 2*πn*, leading to the generalized Smith–Purcell (GSP) condition

6 $$\sin {\theta }_{n\ell }=\frac{1}{\beta }-\left(n-\frac{\ell }{N}\right)\frac{\lambda }{a}$$

in the *ϕ* = 0 plane. This expression is fully general, as it directly applies to any periodic array of scatterers, regardless of its individual elements. Specifically, when compared with the standard SP emission in Eq. (3) for a uniform array, the GSP condition in Eq. (6) is modified by the emergence of the parameter *ℓ* = 0, ⋯ , *N* − 1. When all dipole elements in the array are equally polarized, only the term *ℓ* = 0 survives upon evaluating Eq. (4), which reconciles Eqs. (3) and (6), and reveals that traditional SP is the particular case of GSP arising under such conditions (with all other channels unavailable). However, when the response of the array is non-uniform, Eq. (6) reveals that new channels emerge as possible directions of constructive interference CL emission for each combination of *n* and *ℓ* that fulfills $| \sin {\theta }_{n\ell }| \le 1$, as controlled by the system parameters *β* = *v*/*c*, *a*/*λ*, and *N*. Nevertheless, the polarization considerations described above for traditional SP emission remain valid in this case.

In Fig. 1c, we graphically represent Eq. (6) for the case of an array with *N* = 51 elements and an electron with velocity *v* = 0.1 *c* (≈2.6 keV). Two different values of the array *a*/*λ* are considered, as marked in the respective legend, with respect to some arbitrary design wavelength *λ*. In blue, we see an array with *a*/*λ* = 0.1, which yields a standard SP emission angle (*ℓ* = 0) of *θ*_1,0_ = 0°, which is represented by the thicker arrow in Fig. 1d. However, for a non-uniform array, the GSP emission analysis of this system reveals that additional emission channels are allowed for *ℓ* = 1–5 (with *n* = 1) and *ℓ* = 46–50 (with *n* = 2), as marked by filled blue dots, thus giving rise to a total of 10 additional emission channels through which the array can radiate, and are represented as arrows outgoing along direction *θ*_*nℓ*_ in Fig. 1d. In orange, we present an additional case where we choose *a*/*λ* = 0.25 such that no constructive interference condition can be met, for any value *n*, when *ℓ* = 0 (i.e., Eq. (3) has no real solutions and thus no traditional SP emission can take place). Nevertheless, we find 16 allowed GSP emission channels (filled orange dots), for *n* = 3 and *ℓ* = 13–38, whose emission direction spans the entire *ϕ* = 0 plane (−90° ≤ *θ*_3,*ℓ*_ ≤ 90^∘^), as visible in Fig. 1e.

### Steering of CL using non-uniform arrays

Remarkably, the GSP condition in Eq. (6) is a purely geometrical property of the array and is independent of the polarization of the individual dipoles ${{\bf{p}}}_{j}^{0}$, which affects the emission properties along a given channel, but not the existence of the channel itself. Parameters *v*/*c*, *a*/*λ*, and *N* fully control the emergence and properties of the GSP channels, as long as the array is periodic and its elements are dipolar in nature. In turn, access to the *ℓ*th emission channel (for any *n*) is controlled by the corresponding *ℓ*th GSP component

7 $${\widetilde{{\bf{p}}}}_{\ell }=\frac{1}{N}\mathop{\sum }\limits_{j}{{\bf{p}}}_{j}^{0}{{\mathrm{e}}}^{-2\pi {\mathrm{i}}j\ell /N}$$

which depends on the distribution of the phase-corrected induced dipole moments ${{\bf{p}}}_{j}^{0}$ across the array, governed by the physical properties of the array elements and all intra-array interactions. This means that, to target a specific emission channel denoted by an {*n*, *ℓ*} pair, the induced dipole moment distribution in the array must be engineered to yield a strong Fourier component along harmonic mode *ℓ*.

We discuss in the next section different ways in which, in practice, one may design the array to achieve a given dipole moment distribution **p**_*j*_ in an experimental setup. For now, we assume that we have full control over such distribution. One simple option to achieve the targeting of a specific individual *ℓ* mode is to design the array such that the induced dipole distribution follows a harmonic dependence, such as

8 $$\frac{| {{\bf{p}}}_{j}(\xi )| }{{p}_{0}}=1+A\sin \left(\frac{2\pi \xi j}{N}\right)$$

whose Fourier transform directly yields ${\widetilde{p}}_{\ell }/{p}_{0}={\delta }_{\ell ,0}+A({\delta }_{\ell ,\xi }-{\delta }_{\ell ,N-\xi })/2{\rm i}$, with ${\widetilde{p}}_{\ell }=| {\widetilde{{\bf{p}}}}_{\ell }|$. Here, *ξ* is an integer parameter that represents the frequency of modulation of the dipole moment distribution, which oscillates around the baseline value *p*_0_ with some amplitude ∣*A*∣ ≤ 1. The nonzero baseline of the distribution enforces that the *ℓ* = 0 mode is available for any value of *ξ*, and therefore, traditional SP emission is always present (as long as it is kinematically allowed). However, by choosing the value of *ξ* > 0, we are able to additionally access the GSP modes with *ℓ* = *ξ* and *ℓ* = *N* − *ξ*, and hence the system can radiate along angles *θ*_*n*,*ξ*_ and *θ*_*n*,*N*−*ξ*_ (if the corresponding channel is available for the specific parameters of the array and electron).

**Fig. 2:**
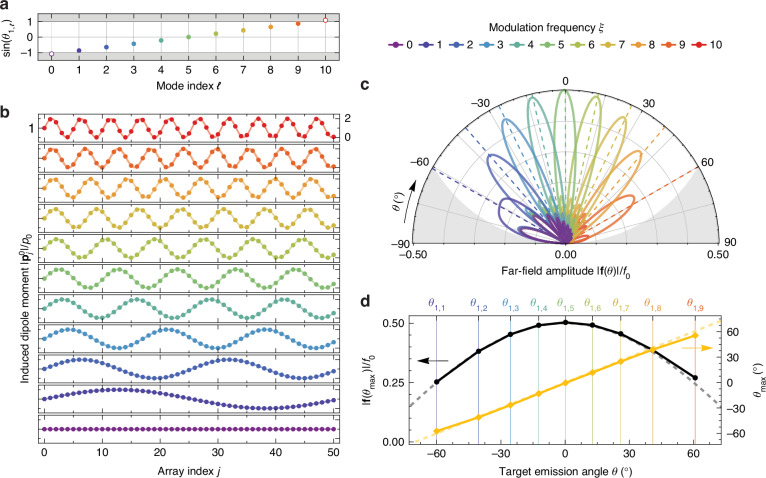
CL steering with non-uniform arrays. **a** GSP condition in Eq. (6) for *n* = 1 and *ℓ* = 0–10 in an array with *N* = 51 elements and period *a*/*λ*_0_ = 0.09, excited by an electron beam with velocity *v* = 0.1 *c* (≈2.6 keV). Modes *ℓ* = 1–9 lie within the region $| \sin {\theta }_{1,\ell }| \le 1$, and are marked by filled circles, whereas modes *ℓ* = 0 and *ℓ* = 10 lie outside that region and are marked with empty circles. Each mode is targeted by the same-color induced dipole moment distribution in (**b**) that selects the mode *ℓ* = *ξ*. **b** Induced dipole moment distributions following Eq. (8) (with oscillation amplitude *A* = 1) for the color-coded values of *ξ* indicated by the legend, as a function of array element index *j*. Each distribution of induced dipole moment $| {{\bf{p}}}_{j}^{0}| /{p}_{0}$ oscillates around 1 between 0 and 2 with a frequency *ξ*. **c** Angle-resolved CL far-field emission amplitude ∣**f**(*θ*)∣ polar plot, normalized to *f*_0_ = *Nk*^2^*p*_0_, as a function of the *θ* angle in the *ϕ* = 0 plane, for an electron passing above an array whose induced dipole moments are given by the same-color curve in (**b**) and polarized along *x* (${{\bf{p}}}_{j}^{0}\parallel \hat{{\bf{x}}}$). The colored dashed lines mark the position of the target angles *θ*_1,*ξ*_ for *ξ* = 1–9, and the shaded gray area represents the condition $| {\bf{f}}(\theta )| /{f}_{0} > \cos \theta$. **d** Peak emission angle *θ*_max_ (right axis) and corresponding peak far-field amplitude ∣**f**(*θ*_max_)∣/*f*_0_ (left axis) as a function of the target emission frequency *θ* for *ξ* = 1−9 (see top axis). The dashed gray curve represents the function $| {\bf{f}}(\theta )| /{f}_{0}=\cos (\theta )/2$, and the yellow dashed line corresponds to the condition *θ*_max_ = *θ*

In what follows, we apply the GSP formalism to steer CL for an electron beam passing near a periodic array. Although SP emission is inherently broadband, we start by designing the array response for a chosen working wavelength *λ*_0_ to illustrate the steering mechanism. We then discuss how the same structure redistributes the emission at other wavelengths according to the GSP condition in Eq. (6). We consider a periodic array with *N* = 51 scatterers and period *a*/*λ*_0_ = 0.09, and an electron moving with velocity *v* = 0.1 *c*. The available radiative GSP modes for these parameters correspond to *ℓ* = 1 − 9 and *n* = 1, as shown in Fig. 2a, yielding emission angles between ≈ ± 60^∘^ distributed roughly symmetrically around the mode *ℓ* = 5, which emits approximately normally to the array. It is important to note that we purposely choose *a*/*λ*_0_ to exclude the *ℓ* = 0 channel and therefore remove the persistent signature of traditional SP. To target each mode, we adopt an induced dipole moment distribution with the form of Eq. (8), with the parameter *ξ* varying from 0 to 10, giving rise to the distributions shown in Fig. 2b. While Eq. (8) prescribes solely the amplitude of each dipole moment, we arbitrarily take all dipoles to be polarized along *x* (that is, ${{\bf{p}}}_{j}=| {{\bf{p}}}_{j}| \hat{{\bf{x}}}$). We discuss the implications of the dipole moment orientation below.

For each dipole moment distribution, we compute the corresponding far-field emission **f**(*θ*) at wavelength *λ*_0_ using Eq. (2), normalized to the reference value *f*_0_ = *Nk*^2^*p*_0_, as a function of the polar angle *θ* and in the *ϕ* = 0 plane, as shown in Fig. 2c. We present only emission along the positive *z* direction (−90° ≤ *θ* ≤ 90°), since emission along the negative *z* axis follows symmetrically due to the properties of the sine function in Eq. (6). The curves for *ξ* = 0 and *ξ* = 10 correspond to cases with no viable GSP emission channel, and thus, there is no pronounced emission at a specific angle. Nevertheless, small residual lobes appear because the array has a finite number of elements *N*, which prevents total destructive interference. In contrast, for *ξ* = 1–9, we observe clear lobes indicating a resonance in the angular emission at well-defined and nearly equally spaced angles ranging between ≈ ± 60°, matching very closely the targeted angles *θ*_1,*ξ*_ marked by dashed lines. This behavior is clearer in Fig. 2d, where we plot the peak far-field amplitude (left axis) and the observed peak emission angle (right axis) as a function of the targeted angle *θ*_1,*ξ*_ for each *ξ* = 1–9. The agreement between target and observed angles is very remarkably accurate, with small deviations (most evident at the smallest and largest targeted angles) stemming from the finite number of array elements (see discussion below). Furthermore, we observe that, in all cases, the peak amplitude follows very closely a $| \cos \theta |$ envelope (dashed gray line) with respect to the theoretical maximum *f*_0_/2 [where the 1/2 comes from the Fourier transform of Eq. (8)]. This envelope, arising from the $| (1-\hat{{\bf{r}}}\otimes \hat{{\bf{r}}})\cdot \hat{{\bf{x}}}|$ term in Eq. (2) for *x*-oriented dipoles, promotes emission into the normal direction (*θ* = 0°) while suppressing emission into the grazing directions (*θ* = ± 90°) in the *ϕ* = 0 plane, similarly to the behavior of a single *x*-polarized point dipole.

**Fig. 3:**
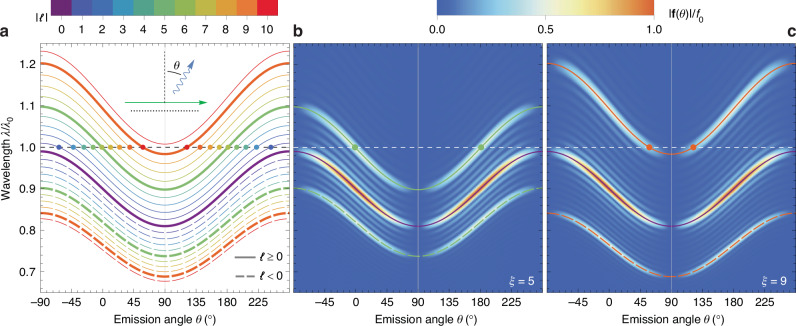
Spectral dependence of GSP emission. **a** Relation between wavelength *λ* and emission angle *θ* for GSP modes labeled by −10 ≤ *ℓ* ≤ 10 (see legends) and *n* = 1. The electron velocity is *v* = 0.1*c*, and we fix *a*/*λ*_0_ = 0.09 and *N* = 51. The curves corresponding to *ℓ* = 0, ± 5, ± 9 are highlighted to facilitate the comparison with (**b**, **c**). The black dashed line corresponds to *λ* = *λ*_0_ (i.e., the same condition as in Fig. 2), and the dots mark its intersection with each color-coordinated curve. The inset shows angle *θ* of light emission (blue arrow) relative to the array (black dots) and electron trajectory (green arrow). **b**, **c** Far-field emission amplitude ∣**f**(*θ*)∣, as a function of *θ* and *λ*, normalized to *f*_0_ = *Nk*^2^*p*_0_, for an array with a dipole moment distribution following Eq. (8) using modulation parameters (b) *ξ* = 5 and (c) *ξ* = 9. In each case, we faintly overlay the same curves and circular dots as in (**a**), corresponding to *ℓ* = 0, ± *ξ*

While in Fig. 2 we explore the optimization of GSP emission at a specific wavelength *λ*_0_, we present the CL emission spectrum in Fig. 3 by sweeping wavelengths *λ* around *λ*_0_ and analyzing their far-field emission profile, keeping *N* = 51, *v* = 0.1*c*, and *a*/*λ*_0_ = 0.09 (same as in Fig. 2). In Fig. 3a, we present the solution of Eq. (6) as a function of wavelength and polar emission angle at the *ϕ* = 0 plane, for modes − 10 ≤ *ℓ* ≤10 and *n* = 1 (see legend). The purple line, corresponding to *ℓ* = 0, is the well-known “S-shaped” curve associated with traditional SP emission^24,28,29^, whereas the different values of *ℓ* generate replicas of this curve at nearby wavelengths. A black dashed line marks the particular case *λ* = *λ*_0_ for which the same conditions as in Fig. 2 are met, and therefore, the intersection of each colored curve with the black dashed line, marked by color-coordinated circles, corresponds to the GSP emission angles marked in Fig. 2c, d (only for modes *ℓ* = 1–9). As the emission wavelength *λ* moves away from *λ*_0_, the accessible modes for each frequency change accordingly: by increasing (reducing) the wavelength, modes with larger (smaller) *ℓ* start to emit. Likewise, although not shown in the figure, when decreasing the wavelength by factors of *n*, the modes with *n* > 1 emerge as well, similarly to traditional SP emission. Finally, as anticipated above, Fig. 2a shows that emission occurs symmetrically for the top and bottom hemispheres.

In Fig. 3b, c, we plot the far-field emission profile for *x*-oriented dipole moment distributions following Eq. (8) with *ξ* = 5 and *ξ* = 9, respectively, and *A* = 1. As discussed above, setting a specific value of *ξ* in Eq. (8) is equivalent to targeting the GSP modes *ℓ* = ± *ξ*, besides the persistent *ℓ* = 0 mode. In agreement with this expectation, in Fig. 2b, c, we observe a strong directional emission precisely for the modes associated with *ℓ* = ± *ξ* and *ℓ* = 0 (note the respective curves/dots in Fig. 3a overlaid on the density maps for clarity). Elsewhere, emission is virtually zero due to destructive interference, except for some fringes attributed to the finite number of dipoles in the array (*N* = 51), which are also observed in Fig. 2c. As discussed above, the plots in Fig. 3b, c also show that emission is modulated by a $\cos \theta$ function, and we note that the far-field amplitude for modes *ℓ* = ± *ξ* is half of the *ℓ* = 0 one due to the fact that ${\widetilde{p}}_{\ell \ne 0}/{\widetilde{p}}_{0} =1/2$, upon Fourier-transforming Eq. (8).

The number of array elements *N* emerges as a crucial parameter determining both the CL steering range and angular resolution. The larger *N* is, the more kinetically-allowed GSP modes exist, and they sample more compactly the kinetically allowed region $| \sin {\theta }_{n\ell }| \le 1$. This results in narrower and more closely packed lobes when increasing the steering range, as shown in Figs. S1 and S2 of the Supplementary Information (SI), which present analogous analyses to those of Figs. 2 and 3 for an array with *N* = 101 elements (instead of *N* = 51). As shown in Figs. S1a and S2a, the larger array supports more viable GSP modes, resulting in a consequent reduction in inter-mode spacing. Furthermore, as we show in Figs. S1b and S2b, c, as the number of discrete steering lobes increases, they simultaneously become narrower, more closely-spaced, and match more accurately the target angles, while the far-field amplitude remains bounded by the $\cos \theta$ envelope (see also Fig. S1d). The steering range for *N* = 101 spans approximately − 70^∘^ < *θ* < 70^∘^, which, however, represents only a mild improvement when compared with *N* = 51. This small increase when approximately doubling *N* is explained by the large slope of the $\arcsin (x)$ function near *x* = ± 1, which needs increasingly larger *N* to reach steering along more grazing angles (closer to ±90°). In Fig. S1d, we show similar results as in S1(b) but for dipoles oriented along *z*. While the available steering angles remain the same (they are independent of the specific distribution **p**_*j*_), the corresponding CL far-field amplitude at the plane *ϕ* = 0 follows now an envelope $| (1-\hat{{\bf{r}}}\otimes \hat{{\bf{r}}})\cdot \hat{{\bf{z}}}| =| \sin \theta |$ (see also Fig. S1e) that suppresses emission into the normal direction and benefits emission along grazing angles. However, the inherent challenge in opening channels for emission at grazing angles discussed above makes *z*-oriented dipoles less suitable for wide-range CL steering. Instead, array elements designed to exhibit a dominant dipolar response along the *x* direction should be more efficient for such a task.

In Figs. S3 and S4 in the SI, we highlight additional properties of the GSP emission. Firstly, Fig. S3(a) shows that the modulation amplitude *A* in Eq. (8) directly controls the far-field peak amplitude, with a larger modulation leading to stronger steering (i.e., larger peak amplitude), but not altering the emission angle. Secondly, Fig. S3b addresses the case where the induced dipole moment distribution is characterized by a superposition of several *ξ* values, which, by the linearity of the Fourier transform, results in the simultaneous emission of CL along each mode *ξ* in the superposition. Then, the associated far-field amplitude along each mode becomes, in that case, proportional to the corresponding superposition coefficient. In Fig. S4, we further show that a random distribution of dipoles behaves, on average, similarly to a uniformly distributed one, showing no preferred direction of emission other than the *ℓ* = 0 one. All of these properties suggest that highly complex emission patterns can be achieved by engineering the induced dipole moment distribution in suitable manners that incorporate different harmonic frequencies *ξ* and amplitudes *A*, and possibly combine dipoles polarized along the *x* and *z* directions, thereby enabling tailored angular responses without modifying the underlying array geometry.

Finally, although the results above were derived for an array of point-like (0D) scatterers, they extend directly to one-dimensional dipole lines that run indefinitely in the *y* direction. As detailed in the “Methods” section and SI, the induced dipole on line *j* can be expanded as **p**_*j*,*q*_, labeled by the longitudinal wave vector *q* arising from the translational invariance of the system along the *y* direction. This dipole component is excited by the same component of the external electric field given by Eq. (10), where the angles (*θ*, *ϕ*) of the far-field CL are identical to the point-dipole result once the component with $q=k\sin \theta \sin \phi$ is selected. Consequently, all design rules in this section are readily adapted to 1D geometries (such as wires or ribbons), as we explore in the next section.

### Engineering non-uniform induced dipole distributions

Up to this point, we have focused on describing the GSP effect and its applications, starting from a finite array with an induced dipole moment distribution presumed to have been previously engineered. Now, we turn our attention to the ways in which one can engineer such arrays in practice, and, in particular, we study physical setups that can be actively tuned to achieve a dynamically induced dipole moment distribution.

The dipole moment ${{\bf{p}}}_{j}={{\bf{p}}}_{j}^{0}{{\mathrm{e}}}^{{\mathrm{i}}\omega ja/v}$ induced in a given element of the array, given by Eq. (1), is primarily governed by its polarizability *α*_*j*_, which captures how the element responds to an external field depending on its geometry, material, and electromagnetic environment. Consequently, realizing a prescribed distribution set {∣**p**_*j*_∣} reduces to inverse-designing a compatible set {*α*_*j*_} that self-consistently generates it. Although one could employ numerical or machine-learning frameworks for this step, we present in the “Methods” section an analytical simplification that yields closed-form polarizability prescriptions under certain conditions. The resulting design is then implemented in the array by choosing the physical properties of each scatterer, either *passively* –by modifying the element geometry (length, width, thickness), material composition or doping (hence its dispersion), and the surrounding electromagnetic environment (substrate permittivity, spacer thickness, cavity backing)—or *actively*—via dynamic tuning such as electrostatic gating, optical/thermal pumping, or voltage biasing.

Here, we focus mainly on active tuning, as we envision that it allows for on-demand control of the array properties that, combined with the results from the previous section, could be applied to realize active steering of CL radiation, among many other technologically-appealing possibilities. Furthermore, we focus on systems composed of 2D or quasi-2D materials naturally exhibiting a dominant in-plane polarization and negligible out-of-plane counterpart, which simplifies the inverse-design process (see “Methods” section) and is more suitable for tunable emission of radiation at near-normal angles, as discussed above. Specifically, we consider arrays of thin VO_2_ disks whose permittivity can be adjusted by controlled fluence exposure, and arrays of monolayer graphene ribbons whose Fermi level can be tuned by electrostatic gating (see “Methods” section). Importantly, we note that the external stimuli that we employ in these examples exclusively control the state of the individual array elements (i.e., VO_2_ phase and graphene Fermi level), but do not supply gain or a phase-matched input to the emitted field. Therefore, the resulting CL emission remains a spontaneous free-electron process rather than stimulated radiation. In both types of systems, we preserve the array number of elements *N* = 51, electron velocity *v* = 0.1 *c* (≈2.6 keV), and period-to-wavelength ratio *a*/*λ*_0_ = 0.09 chosen in Fig. 2 and, as such, the allowed GSP emission channels (*n* = 1, *ℓ* = 1–9, see Fig. 2a) remain unchanged. As examples, we reverse-design the fluence dosage per disk and the Fermi level per ribbon necessary to imprint in the array an induced dipole moment distribution given by Eq. (8) with *ξ* = 3 and *ξ* = 7, yielding a target emission along angles *θ*_1,3_ = −25.5° and *θ*_1,7_ = 26.0°.

#### VO_2_ disks

Vanadium dioxide (VO_2_) is a phase-changing material that undergoes a rapid, reversible insulator–metal transition (IMT) at a modest temperature (~340 K)^34,35^. Such a transition is accompanied by an abrupt change in optical and electrical properties, with VO_2_ exhibiting markedly different dielectric functions at the insulating and metallic phases^36,37^, as shown in Fig. S5a in the SI. Nevertheless, intermediate states in between the two phases can be accessed by controlling the temperature of the material, and are typically parameterized by a metallic volume fraction *f*_m_ ranging between 0 and 1 (see “Methods” section). As the material cools down, it reverts slowly to the insulator phase, with a characteristic time scale that depends on how quickly the structure releases heat, ranging from tens to hundreds of nanoseconds in thin films on substrates^38,39^ to microseconds in suspended or weakly coupled nanostructures^40^. VO_2_ further counts with mature thin-film growth and integration on common substrates^41^, and reliable nanofabrication with reversible optical/electrical/thermal switching demonstrated from ultrafast to microsecond regimes^34,35,38,40,42,43^, which make it a suitable candidate for active switching applications.

**Fig. 4:**
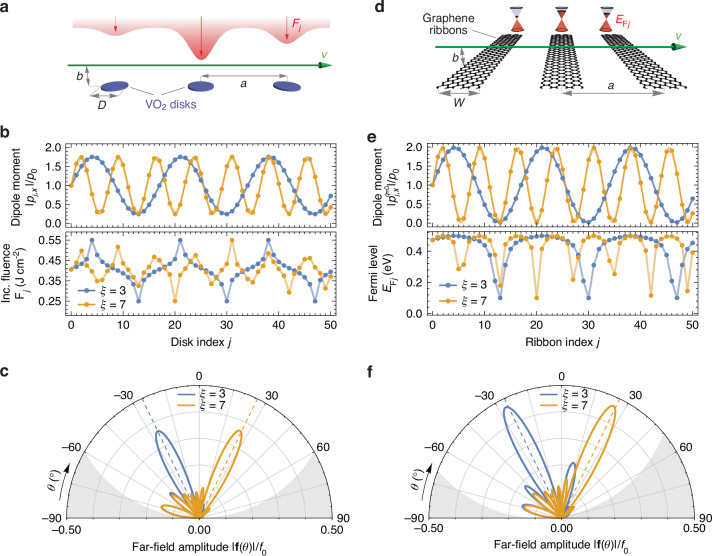
Active tuning of CL emission. **a** Scheme of an array of VO_2_ disks with diameter *D* = 250 nm and thickness *t* = 2 nm, separated from their nearest neighbors by a center-to-center distance *a* = 450 nm, with an electron passing parallel to the array at a distance *b* = 10 nm and with velocity *v* = 0.1 *c*. The array is illuminated by a pump beam (in red) that is spatially engineered to deliver a fluence *F*_*j*_ at the *j*th array element. **b** Induced dipole moment at a wavelength *λ*_0_ = 2*πc*/*ω* = 5.0 *μ*m (top) along different array elements, following Eq. (8) with *ξ* values as indicated by the labels, *A* = 1, and *p*_0_ = 5.4 × 10^−3^(*eD*/*ω*), as a function of element *j*, for the color-coordinated fluence distributions (bottom), ranging between *F*_*min*_ = 0.25 J cm^−2^ and *F*_*max*_ = 0.55 J cm^−2^. **c** Far-field emission distribution for the same-color array distributions in (**b**), with *f*_0_ = 2.7 × 10^−2^(*e*/*Dω*). **d**–**f** Same as (**a**–**c**), but for an array of graphene ribbons with width *W* = 100 nm whose *j*th element’s Fermi level is set to *E*_F*j*_ (as depicted by the Dirac cones) between ${E}_{F,\min }=0.1$ eV and ${E}_{F,max}=0.5$ eV, with a period *a* = 615 nm and evaluated at a wavelength *λ*_0_ = 6.8 *μ*m. In (**e**), the dipole moment corresponds to the *q* = 0 component, with *p*_0_ = 4.48(*eW*/*ω*). In **f**, we have *f*_0_ = 1.95/(*e*/*Wω*)

Figure 4a illustrates an array of VO_2_ disks with a diameter of *D* = 250 nm, thickness of *t* = 2 nm^41^, and period *a* = 450 nm. The structure is illuminated by an incident pump optical pulse whose wavefront is engineered to deliver specific energy doses to different disks, thereby inducing spatially varying local temperatures. As a result, each disk experiences a distinct optical excitation fluence *F*_*j*_, leading to a controlled modification of its permittivity through the thermally driven IMT in VO_2_. The variation in the polarizability of such disks with incident fluence at the target probe wavelength *λ*_0_ = 5.0 *μ*m (chosen to yield a large contrast between the insulating and metallic phases of the VO_2_) is shown in Figure S6a in the SI (see also “Methods” section) for a pump laser with wavelength *λ*_pump_ = 632 nm. In practice, such modulation of the excitation can be customized using a spatial light modulator, controlled interference of multiple beams, or active metamaterial masks, provided that the required spatial pattern does not contain features below the optical diffraction limit of the pumping wavelength (i.e., if *a* ≳ *λ*_pump_).

Under illumination by an electron beam with impact parameter *b* = 10 nm, as also shown in Fig. 4a, the interference pattern of the emitted CL depends on the fluence dose delivered to each disk. We note that the electron traverses the structure in a timescale *Na*/*v* of the order of a few picoseconds, during which the permittivity of the disks can be regarded as approximately constant. In Fig. 4b (bottom panel), we show two distinct fluence-delivery patterns that were inverse-designed as described in the “Methods” to generate, using Eqs. (1) and (9), the dipole moment distributions in the color-coordinated curves in the top panel. As warranted, the reconstructed induced dipole moment distribution successfully reproduces Eq. (8) with *ξ* = 3 (blue) and *ξ* = 7 (orange). Although dipole-dipole interactions were considered for the reconstruction algorithm (see “Methods” section), the relatively small dipole moment in each disk and the large separation between consecutive disks made them negligible.

Using Eq. (2), we finally calculate the resulting far-field emission patterns, displayed in Fig. 4c, confirming that the emitted radiation is strongly concentrated around the desired target angles *θ*_1,3_ and *θ*_1,7_, demonstrating excellent agreement between the designed and realized responses. The other available emission angles for the considered geometrical parameters (*ξ* = 1–9, as in Fig. 2) can be targeted analogously. We note that the emission amplitude falls short of its theoretical maximum value *f*_0_/2 due to a finite background dipole moment arising from the weakly polarizable insulating phase. As a result, although the metallic phase exhibits a substantially larger polarizability, the modulation depth *A* of the dipole amplitude in Eq. (8) cannot reach unity (*A* ≈ 0.75 for the example in Fig. 4b).

#### Graphene ribbons

Graphene nanoribbons constitute a highly versatile alternative platform for implementing the type of spatially controlled optical modulation of CL. Their optical and electronic properties can be tuned continuously through electrostatic gating, chemical doping, or strain, offering direct control over the charge carrier density and consequently the induced dipole moment^44,45^. In contrast to confined structures such as nanodisks, ribbons support extended plasmonic modes with well-defined momentum along their length, which can be exploited to engineer collective responses^46,47^. Furthermore, graphene ribbons are particularly well suited for experimental realization, since they can be fabricated with high precision using established lithographic and transfer techniques^48,49^, integrated into a variety of substrates^50,51^, and interfaced readily with local gate electrodes^52,53^. With suitable device design^54^, the Fermi level of each ribbon can be individually adjusted, enabling spatially resolved and actively reconfigurable modulation patterns. These attributes make graphene nanoribbons an attractive and practical platform for dynamic control of light–matter interactions.

In Fig. 4d, we present an example of an array of graphene nanoribbons with a width of *W* = 100 nm and a period of *a* = 615 nm. The ribbons in this array are assumed to be individually tunable, allowing independent control of their Fermi levels. We target an optical wavelength of *λ*_0_ = 6.8 *μ*m (≈0.18 eV) corresponding to the resonance wavelength for a 0.5-eV-doped graphene ribbon, as shown in Fig. S6b in the SI. Figure 4e (bottom) illustrates two representative Fermi-level profiles for an array comprising *N* = 51 ribbons, ranging between 0.1 and 0.5 eV, which are designed to produce the dipole-moment distributions shown in the top panel following Eq. (8), with *ξ* = 3 (blue) and *ξ* = 7 (orange). We note that such a dipole moment corresponds to the *q* = 0 component (see SI) that controls the GSP emission along the *ϕ* = 0 plane, as discussed above. As in the disk's geometry, dipole-dipole interactions were included in the inverse design algorithm, but turned out to be negligible for the chosen geometric parameters. However, in contrast to the disks, here the modulation depth *A* can approach unity, as the polarizability of each graphene ribbon is strongly dependent on its doping level and nearly vanishes when off-resonance. Figure 4f displays the corresponding far-field emission patterns, demonstrating that the radiated amplitude is concentrated around the desired target angles *θ*_1,3_ and *θ*_1,7_, in excellent agreement with the designed emission directions.

#### Alternative passive methods

In the SI, we present for completeness two examples of passive engineering, which involve adjusting the length of small silver nanorods and the width of thin gold ribbons, whose polarizability can be controlled through their dimensions as shown in Fig. S6 in the SI. As above, we consider arrays with *N* = 51 elements, a ratio *a*/*λ*_0_ = 0.09, and *v* = 0.1 *c*. While these structures are not actively tunable, they have the advantage of being conceptually simpler and potentially easier to realize. In Fig. S7, we show the inverse design of the physical dimensions of each scatterer, leading again to a highly directional emission of light that follows the target angles for each distribution closely. Interestingly, the large dipole moment achieved by the noble metals considered in these examples renders the dipole-dipole interactions non-negligible, but the procedure explained in the “Methods” section is able to account for those successfully, as proven by the reconstructed dipole moment distribution.

Observing the designed length profile of the nanorods, one notes that reproducing the theoretically prescribed geometry could demand high fabrication precision. This is because even a small deviation in length can lead to a significant change in the rod polarizability (see Fig. S6 in the SI), thereby affecting the intended dipole distribution. While this effect is particularly pronounced for silver nanorods, it also applies to gold and graphene nanoribbons, where the response relies on the strong dipolar resonances supported by these structures. Nevertheless, this challenge can be mitigated by inverse-designing the polarizability in a regime where its dependence on the control parameter (length, width, or Fermi level) is smoother (i.e., slightly off-resonant) or by targeting broader, lower-quality-factor resonances. Such approaches should relax fabrication tolerances at the cost of a reduced induced dipole amplitude and, consequently, lower light-emission efficiency, which can, however, be compensated by adjusting the intensity or impact parameter of the electron beam.

## Discussion

We have introduced a generalized framework for SP radiation in finite and non-uniform arrays, extending the classical concept to structures with spatially varying dipole moments. This GSP condition enables emission into non-traditional angles and spectral channels, whose accessibility is determined by the engineered amplitude and phase distribution of the dipoles. By prescribing sinusoidal modulations of the dipole moment across the array, we have demonstrated the ability to steer CL emission actively and predictably, thereby establishing a versatile approach to programmable free-electron light sources. Nevertheless, our concept and methods can be straightforwardly generalized to other types of modulation profiles.

Two representative active implementations were presented: arrays of VO_2_ nanodisks and graphene nanoribbons. In VO_2_, patterned optical excitation enables spatial modulation of the local permittivity through thermally driven IMTs, while in graphene, the dipole response can be tuned continuously through electrostatic gating. Both systems achieve targeted angular emission in agreement with the GSP theory. The combination of established materials and accessible tuning mechanisms makes these predictions directly testable in angle-resolved CL experiments. We find that graphene offers nearly complete modulation depth owing to its highly controllable polarizability. Collectively, these results highlight the remarkable degree of control attainable with actively tunable materials whose polarizability can be controlled in situ. By adjusting the optical properties of the array elements in real time, one can switch between different excitation profiles and, consequently, between distinct dipole-moment distributions for tailoring free-electron-driven light emission. Increasing the number of array elements correspondingly increases the degrees of freedom available for realizing more complex emission patterns. Some additional degree of tunability can still be achieved by adjusting the velocity *v* and impact parameter *b* of the electron beam.

Beyond the examples presented in this paper, a broad range of alternative materials and mechanisms may be harnessed to realize GSP modulation, including thermal or electrostatic tuning of transition-metal dichalcogenides (TMDs), phase-change chalcogenides, or photo-doped 2D materials such as graphene and MoS_2_. Alternatively, instead of tuning the emitters themselves, one could control the surrounding environment using active substrates that can be spatially modulated, thereby enabling additional degrees of reconfigurability. Together, these possibilities suggest multiple realistic experimental routes to implement and benchmark GSP steering across different material platforms.

The formalism developed in this work is general and can be extended beyond the electrostatic approximation used for analytical traceability of the scatterers. While we have modeled each scatterer as a point (or line) dipole exhibiting a quasistatic response, the central features of the GSP condition and the resulting directional emission should remain valid when retardation and finite-size effects are taken into account, albeit the precise resonance conditions may shift slightly. Such effects primarily introduce quantitative corrections to the inverse-design procedure, which can be compensated for by appropriate adjustments of the tuning parameters, without altering the qualitative concept explored here. Moreover, although the present work focuses on effectively one-dimensional arrays consistent with the geometry defined by a single electron trajectory, the framework readily extends to more complex excitation schemes, including multi-beam or 2D arrangements. In practice, this corresponds to extending the 1D array to a 2D lattice (e.g., a square array of scatterers), for which the GSP condition becomes vectorial (i.e., the phase-matching condition involves two in-plane components owing to the additional periodicity of the array) and, for that reason, unlocks independent control over more degrees of freedom, such as both polar and azimuthal emission angles.

Although we illustrate our approach using VO_2_ and graphene at representative mid-infrared wavelengths, the GSP framework is broadly applicable across the electromagnetic spectrum, provided suitable resonant scatterers and tuning mechanisms are available, and the required geometries are within practical fabrication limits. SP emission can thus serve as a viable radiation source, complementing mature technologies that rely on fundamentally different operating principles (e.g., quantum cascade lasers or optical parametric sources in the mid-IR) through its natural compatibility with electron-beam systems, broadband angle–wavelength selectivity, and, crucially, reconfigurability, which enables on-demand steering and shaping of selected spectral components.

In summary, the results presented in this work highlight a powerful strategy for bridging near-field electron excitation with metasurface design. By tailoring the local response of individual elements, one can dynamically reconfigure the collective radiation pattern without altering the underlying geometry. Such control opens new possibilities for reconfigurable free-electron light sources, adaptive beam steering, spectral shaping, and on-demand generation of structured or directional emission. In the longer term, this approach may enable electron-driven nanophotonic devices for active holography, ultrafast sensing, and programmable quantum light generation.

## Materials and methods

### Field generated by a moving electron

We consider an electron moving along a trajectory defined by *y* = 0 and *z* = *b*, with constant velocity *v* along *x* (see Fig. 1b). The electric field generated by such an electron at frequency *ω* and position ${\bf{r}}=x\hat{{\bf{x}}}+{\bf{R}}$ is given by^12^

9 $${{\bf{E}}}^{{\mathrm{ext}}}({\bf{r}})=\frac{2e\omega }{{v}^{2}\gamma }\left[\frac{{\rm i}}{\gamma }{K}_{0}\left(\frac{\omega R}{v\gamma }\right)\hat{{\bf{x}}}-{K}_{1}\left(\frac{\omega R}{v\gamma }\right)\hat{{\bf{R}}}\right]{{\mathrm{e}}}^{{\mathrm{i}}\omega x/v}$$

where the spatial coordinates are defined as ${\bf{R}}=y\hat{{\bf{y}}}+(z-b)\hat{{\bf{z}}}$, *R* = ∣**R**∣, $\hat{{\bf{R}}}={\bf{R}}/R$, and the Lorentz factor $\gamma =1/\sqrt{1-{v}^{2}/{c}^{2}}$.

To describe translationally-invariant systems along *y* (e.g., ribbons) illuminated by the electron, it is convenient to expand the electric field as ${{\bf{E}}}^{\mathrm{ext}}({\bf{r}})={(2\pi )}^{-1}\int {\rm{d}}q\,{{\bf{E}}}_{q}^{\mathrm{ext}}(x,z){{\rm{e}}}^{\mathrm{iqy}}$, where *q* is the wave vector along *y*, and

10 $${{\bf{E}}}_{q}^{{\mathrm{ext}}}(x,z)=\frac{2\pi {\mathrm{i}}e}{v{\kappa }_{z}}\left(\frac{\omega }{v{\gamma }^{2}},q,{\rm{i}}{\kappa }_{z}\right){{\mathrm{e}}}^{-{\kappa }_{z}| z-b| }{{\mathrm{e}}}^{{\mathrm{i}}\omega x/v}$$

and we introduce ${\kappa }_{z}^{2}={\omega }^{2}/{v}^{2}{\gamma }^{2}+{q}^{2}$.

### Generalized SP effect with 1D scatterers

We extend the GSP condition for point dipoles to an array of line dipoles placed periodically along *x*, with a period *a*, infinitely extended along *y*, and located in the plane *z* = 0. In practice, each line can represent a ribbon or cylinder that is sufficiently narrow and well separated from its neighbors such that, in the far field, it can be approximated by a line with negligible lateral extent. As above, we take the electron moving parallel to *x* with velocity ${\bf{v}}=v\hat{{\bf{x}}}$ along a trajectory defined by *y* = 0 and *z* = *b*.

Unlike the point dipole structure, now the line *j* exhibits a dipole moment density **P**_*j*,*y*_ (per unit length along *y*), which depends on the coordinate *y*. Following the same procedure as above, we anticipate that the finite electron velocity leads to a phase difference to be imprinted on the dipole moment induced in each line, so we write ${{\bf{P}}}_{j,y}={{\bf{P}}}_{j,y}^{0}{{\mathrm{e}}}^{{\mathrm{i}}\omega ja/v}$. Then, the differential far-field amplitude generated by a dipole element d*y* at *y* can be written analogously to Eq. (2) as

11 $${\rm d}{\bf{f}}(\hat{{\bf{r}}})={k}^{2}(1-\hat{{\bf{r}}}\otimes \hat{{\bf{r}}})\cdot \mathop{\sum }\limits_{j}{{\bf{P}}}_{j,y}^{0}{\rm e}^{{\rm i}kja/\beta }{\rm e}^{-{\rm i}k({{\bf{r}}}_{j}\cdot \hat{{\bf{r}}})}{\rm d}y$$

where **r**_*j*_ = (*ja*, *y*, 0), and the total far field follows as ${\bf{f}}(\hat{{\bf{r}}})=\int {\mathrm{d}}y\,[{\mathrm{d}}{\bf{f}}(\hat{{\bf{r}}})/{\mathrm{d}}y]$.

To simplify the equation above, we note that the translational symmetry of the problem allows us to write, in general, the *y*-dependence of the dipole moment of line *j* in terms of a wave vector *q* as

12 $${{\bf{P}}}_{j,y}=\frac{1}{2\pi }\int {\mathrm{d}}q\,{{\bf{P}}}_{j,q}{{\mathrm{e}}}^{{\mathrm{i}}qy}$$

whose expansion components **P**_*j*,*q*_ represent the eigenmodes of each line element, parameterized by *q*, and whose profiles can be determined depending on its material and geometric parameters (such as the permittivity/conductivity and dimensionality). When arranged in an array with period *a*, the self-consistent induced dipole moment density at each ribbon and for momentum component *q* follows from the self-consistent equation ${{\bf{P}}}_{j,q}={\alpha }_{j,q}\cdot [{{\bf{E}}}_{q}^{{\mathrm{ext}}}(ja,0)+{\sum }_{i\ne j}{{\mathcal{G}}}_{q,ji}\cdot {{\bf{P}}}_{i,q}]$ [i.e., analogous to Eq. (1) for point dipoles], where the *q*-component electron field ${{\bf{E}}}_{q}^{{\mathrm{ext}}}$ is shown above, and the corresponding ribbon polarizability *α*_*j*,*q*_ and line dipole-line dipole interaction Green tensor ${{\mathcal{G}}}_{q,ji}$ are discussed in the SI.

Replacing Eq. (12) into Eq. (11) and performing the integration over *y*, we obtain a term with the form $\int {\rm{d}}y\,{{\mathrm{e}}}^{{\mathrm{i}}qy}{{\mathrm{e}}}^{-{\mathrm{i}}ky(\hat{{\bf{y}}}\cdot \hat{{\bf{r}}})}=2\pi \delta (q-{k}_{y})$, where we define ${k}_{y}=k(\hat{{\bf{y}}}\cdot \hat{{\bf{r}}})=k\sin \theta \sin \phi$. At this point, the Dirac delta function resolves the *q*-integral, yielding the final expression

13 $${\bf{f}}(\hat{{\bf{r}}})={k}^{2}(1-\hat{{\bf{r}}}\otimes \hat{{\bf{r}}})\cdot \mathop{\sum }\limits_{j}{{\bf{P}}}_{j,{k}_{y}}^{0}{{\mathrm{e}}}^{{\mathrm{i}}kja/\beta }{{\mathrm{e}}}^{-{\mathrm{i}}kaj(\hat{{\bf{x}}}\cdot \hat{{\bf{r}}})}$$

Equation (13) is remarkably similar to Eq. (2) (for an array of point dipoles) with the exception that, for a given outgoing direction $\hat{{\bf{r}}}$, we must take only the $q=k(\hat{{\bf{y}}}\cdot \hat{{\bf{r}}})$ component out of the full momentum distribution of **P**_*j*,*y*_ in Eq. (12). In particular, the SP pattern remains exactly the same as before in the *ϕ* = 0 plane when only the *q* = 0 components are considered.

### Inverse design of the polarizability

We aim to map a given phase-corrected induced dipole moment distribution ${{\bf{p}}}_{j}^{0}={{\bf{p}}}_{j}{{\mathrm{e}}}^{-{\rm{i}}\omega ja/v}={p}_{j,x}^{0}\hat{{\bf{x}}}+{p}_{j,z}^{0}\hat{{\bf{z}}}$ onto a polarizability tensor distribution, where each element has components ${\alpha }_{j}^{\nu \mu }$ (*ν*, *μ* = *x*, *y*, *z*). For simplicity, we restrict this analysis to diagonal polarizability tensors obeying the property ${\alpha }_{j}^{\nu \mu }={\alpha }_{j}^{\nu \nu }{\delta }_{\mu \nu }$.

In general, we cannot prescribe both components of the dipole moment, since they are not independent, but must rather preserve the self-consistency of the coupled-dipole formalism with respect to the external field [in this case, the electron beam, whose field is given by Eq. (9)], and the dipole-dipole interaction between the different elements of the array. Furthermore, in general, one may aim to achieve a specific dipole moment profile up to a global scaling constant [eg., *p*_0_ in Eq. (8)] that must be determined self-consistently. From these two considerations, we can write ${p}_{j,\nu }^{0}={p}_{0}{b}_{j,\nu }$, and assume that all elements *b*_*j*,*ν*_ are prescribed for one specific component *ν*. From here, we aim to reconstruct some physically compatible polarizability components ${\alpha }_{j}^{\nu \nu }$, together with *p*_0_ and the remaining induced dipole components.

Introducing ${{\bf{E}}}_{0}^{{\mathrm{ext}}}={{\bf{E}}}^{{\mathrm{ext}}}({{\bf{r}}}_{j}){{\mathrm{e}}}^{-{\mathrm{i}}\omega ja/v}$ (which is uniform across the array) and the notation defined above, we can use Eq. (1) to obtain the relation

14 $${\alpha }_{j}^{\nu \nu }=\frac{{p}_{0}{b}_{j,\nu }}{{E}_{0,\nu }^{{\mathrm{ext}}}+{p}_{0}{\sum }_{i}{\sum }_{\mu }{{\mathcal{G}}}_{ji}^{\nu \mu }{b}_{i,\mu }}$$

While this equation precisely relates the polarizability and self-consistent dipole moment across an array [it is equivalent to Eq. (1)], it is not general enough to reconstruct unequivocally {*b*_*j*,*ν*_} into $\{{\alpha }_{j}^{\nu \nu }\}$ because it yields separate prescriptions for ${\alpha }_{j}^{xx}$ and ${\alpha }_{j}^{zz}$ that must be mutually compatible with the material and geometry chosen and the external field components. Therefore, Eq. (14) (for *ν* = *x*, *z*) must be supplied by additional information regarding the symmetry of the polarizability tensor (e.g., ${\alpha }_{j}^{xx}={\alpha }_{j}^{zz}$ if it is isotropic) to form a nonlinear system of 3*N* equations that can be numerically solved to yield both components of the polarizability tensors and the non-prescribed component of the induced dipole moment. Finally, *p*_0_ can be set by imposing an additional condition: for example, the array element where the induced dipole is maximum (according to the prescription) must exhibit the maximum designed polarizability allowed by our physical system.

While the process described above is, in general, computationally heavy for more than a few array elements, there are two scenarios in which Eq. (14) can be simplified and employed directly:

- When the array elements are sufficiently far apart, their dipole-dipole interactions become negligible, and Eq. (14) simplifies to ${\alpha }_{j}^{\nu \nu }={p}_{0}{b}_{j,\nu }/{E}_{0,\nu }^{{\mathrm{ext}}}$, which decouples the *x*- and *z*-components. Ensuring self-consistency becomes trivial: for an isotropic tensor, for example, where the coefficients *b*_*j*,*x*_ are prescribed, it suffices to set ${b}_{j,z}={b}_{j,x}({E}_{0,z}^{{\mathrm{ext}}}/{E}_{0,x}^{{\mathrm{ext}}})$ and ${p}_{0}={\alpha }_{{\mathrm{max}}}{E}_{0,x}^{{\mathrm{ext}}}/{b}_{{\mathrm{max}}}$, with *b*_max_ denoting the maximum value in the prescribed set {*b*_*j*,*x*_}, and *α*_max_ the maximum polarizability of the array elements.
- If the scatterers are highly anisotropic, such that one component *ν* of the polarizability dominates, while the orthogonal one is strongly suppressed or removed (e.g., in 2D materials), then we can approximate Eq. (14) for the dominant component as ${\alpha }_{j}^{\nu \nu }={p}_{0}{b}_{j,\nu }/({E}_{0,\nu }^{{\mathrm{ext}}}+{p}_{0}{\sum }_{i}{{\mathcal{G}}}_{ji}^{\nu \nu }{b}_{i,\nu })$ and set ${p}_{0}={E}_{0,\nu }^{{\mathrm{ext}}}{({b}_{{\mathrm{max}}}/{\alpha }_{{\mathrm{max}}}-{\sum }_{i}{{\mathcal{G}}}_{ji}^{\nu \nu }{b}_{i,\nu })}^{-1}$, while taking the polarizability (and the dipole moment) along the other direction to be zero. Under such conditions, the equation above leads to an accurate mapping of ${\alpha }_{j}^{\nu \nu }$ without introducing any inconsistency.

### Dipolar response of electrostatic structures

#### Thin nanodisks

The in-plane polarizability of a disk with diameter *D* ≪ *λ* and thickness *t* ≪ *D* composed of a material described by a 2D surface conductivity *σ*(*ω*) at frequency *ω* = 2*πc*/*λ* and embedded in vacuum can be written in the electrostatic limit as^46,55^

15 $$\alpha (\omega )={D}^{3}\mathop{\sum }\limits_{m}\frac{{\zeta }_{m}^{2}}{1/\eta (\omega )-1/{\eta }_{m}}$$

where *η*(*ω*) = i*σ*(*ω*)/*Dω*, and the *m*-sum spans the different multipolar modes supported by the system, parameterized by the eigenvalues *η*_*m*_ and mode dipole moments *ζ*_*m*_. For simplicity, in the examples presented in this paper, we take only the dipolar response (*m* = 1) and use the fits ${\eta }_{1}={a}_{\eta }\exp ({b}_{\eta }x)+{c}_{\eta }$ and ${\zeta }_{1}={a}_{\zeta }\exp ({b}_{\zeta }x)+{c}_{\zeta }$ reported in the literature^55^, where *x* = *t*/*D*. Specifically, for a disk of diameter *D*, we take *a*_*η*_ = 0.03801, *b*_*η*_ = −8.569, *c*_*η*_ = −0.1108, *a*_*ζ*_ = −0.01267, *b*_*ζ*_ = −45.34, and *c*_*ζ*_ = 0.8635.

The absorption of incident radiation by such a disk is described by its corresponding absorption cross section *σ*_abs_(*ω*) = *σ*_ext_(*ω*) − *σ*_sc_(*ω*), written in terms of the extinction ${\sigma }_{{\mathrm{ext}}}(\omega )=4\pi k\,{\mathrm{Im}}\{\alpha (\omega )\}$ and scattering *σ*_sc_(*ω*) = 8*πk*^4^∣*α*(*ω*)∣^2^/3 counterparts (with *k* = *ω*/*c*) valid when *D* ≪ *λ*.

#### Thin nanoribbons

Unlike disks and other finite-size structures, nanoribbons are infinite along their longitudinal direction, and therefore, the associated normal modes are characterized by a wave vector *q*. In the SI, we show that, when exposed to an external field component of transversal wave vector *q*, the ribbon exhibits a dipole moment density **P**_*q*_ associated with an effective polarizability tensor *α*_*q*_ given in Eq. (S9) in the SI. For *q* = 0, as considered in the main text, the response of a dipole can be simply described by its *xx* component with the form

16 $${\alpha }_{0}^{xx}(\omega )={W}^{2}\mathop{\sum }\limits_{m}\frac{{\zeta }_{m0}^{2}}{1/\eta (\omega )-1/{\eta }_{m0}}$$

where *ζ*_1,0_ = 0.942 and *η*_1,0_ = −0.069 for the nanoribbon dipolar *m* = 1 mode (see SI).

#### Nanorods

We model the polarizability of nanorods in vacuum with a permittivity *ϵ*, length *L*, and tip radius of *L*/2*R* (where *R* is the aspect ratio of the rod), in the electrostatic limit (*L* ≪ *λ*), as^56^

17 $$\alpha (\omega )=\frac{1}{4\pi }\mathop{\sum }\limits_{m}{V}_{m}{\left(\frac{1}{\epsilon -1}-\frac{1}{{\epsilon }_{m}-1}\right)}^{-1}$$

where, as above, the *m*-sum spans the multipolar modes of the system. For simplicity, we take only the dipolar mode (*m* = 1), for which we use fitted parameters from the literature^56^, specifically, *V*_1_ = 0.896*V* and *ϵ*_1_ = −1.73*R*^1.45^ − 0.296, with *V* = *πL*^3^(3*R* − 1)/12*R*^3^ being the nanorod volume.

### Material modeling

Here, we show the adopted models to describe the optical response of the different materials considered in this work. For the thin VO_2_ disks and Au ribbons of small thickness *t*, we assume the structures to be effectively 2D, and we describe them through a surface conductivity related to their permittivity *ϵ* by^57^ *σ* = i*ωt*(1 − *ϵ*)/4*π*.

#### Vanadium dioxide

For a given value of the metallic fraction *f*_m_ ranging between 0 and 1, we model the mixed-phase permittivity of VO_2_, ${\epsilon }_{{{\mathrm{VO}}}_{2}}(\omega )$, at frequency *ω*, using the Bruggeman effective-medium relation^37,42,58^

18 $${f}_{{\mathrm{m}}}\frac{{\epsilon }_{{\mathrm{m}}}-{\epsilon }_{{{\mathrm{VO}}}_{2}}}{{\epsilon }_{{\mathrm{m}}}+2{\epsilon }_{{{\mathrm{VO}}}_{2}}}+(1-{f}_{{\mathrm{m}}})\frac{{\epsilon }_{{\mathrm{i}}}-{\epsilon }_{{{\mathrm{VO}}}_{2}}}{{\epsilon }_{{\mathrm{i}}}+2{\epsilon }_{{{\mathrm{VO}}}_{2}}}=0$$

where *ϵ*_i_(*ω*) and *ϵ*_m_(*ω*) are the insulating and metallic phase permittivities of VO_2_^59^, respectively (see Fig. S5b in the SI).

As described in detail in the SI, we model the fluence dependence of *f*_m_ for a VO_2_ disk with diameter *D* = 250 nm and thickness *t* = 2 nm (see Fig. 4) under illumination by a light pulse of fluence *F* and wavelength 632 nm as

19 $${f}_{{\rm{m}}}(F)=\frac{1}{1+\exp [-(F-{F}_{{\mathrm{m}}})/\Delta F]}$$

where *F*_m_ = 0.395 J cm^−2^ and Δ*F* = 0.019 J cm^−2^ (see SI for details). We note that the relatively large fluence necessary to produce mild temperature increases in the disk reflects its very small absorption cross section (see Fig. S5d in the SI).

#### Graphene

We take graphene to be described by an isotropic 2D optical conductivity with the Drude form^44^

20 $$\sigma (\omega )=\frac{{\mathrm{i}}{e}^{2}}{\pi {\hslash }^{2}}\frac{{E}_{{\mathrm{F}}}}{\omega +{\mathrm{i}}{\tau }^{-1}}$$

at frequency *ω*, where *E*_F_ is its Fermi level, $\tau =\mu {E}_{{\rm{F}}}/e{v}_{{\rm{F}}}^{2}$ represents the inelastic scattering time for a certain mobility *μ*, and *v*_F_ ≈ *c*/300 is the Fermi velocity of electrons in graphene. In this work, we take *μ* = 10,000 cm^2^ V^−1^ s^−1^. The expression above contains exclusively the intraband response of graphene and, therefore, is valid for *ℏω* ≲ 2*E*_F_ and *E*_F_ ≫ *k*_B_*T* at a certain electron temperature *T*. While here we choose to use Eq. (20) for the sake of simplicity, a full description of the graphene conductivity including interband transitions, high electron temperature, and/or nonlocal effects can be found in the literature^60^.

#### Noble metals

We model the permittivity of gold and silver, used in Figs. S6 and S7 in the SI, through the Drude model^57^,

21 $$\epsilon (\omega )={\epsilon }_{\mathrm{b}}-\frac{{\omega }_{{\mathrm{p}}}^{2}}{\omega (\omega +{\mathrm{i}}{\gamma }_{{\mathrm{m}}})}$$

where the plasma frequency *ω*_p_, inelastic damping rate *γ*_m_, and background permittivity *ϵ*_b_ are fitted from experimental data^56,61^. Specifically, for gold, we take *ℏω*_p_ = 9.06 eV, *ℏγ*_m_ = 71 meV, and *ϵ*_b_ = 9.5; for silver, we use *ℏω*_p_ = 9.17 eV, *ℏγ*_m_ = 21 meV, and *ϵ*_b_ = 4.0.

## Supplementary information

Supplementary information for Active steering of cathodoluminescence through a generalized Smith–Purcell effect

## Data Availability

All data needed to evaluate the conclusions in the paper are present in the paper and the Supplementary Material. Additional data related to this paper may be requested from the authors.
