# Supplementary material for: Active steering of cathodoluminescence through a generalized Smith–Purcell effect
Source: Light Sci Appl. 2026 May 6;15:218. doi: 10.1038/s41377-026-02280-y (PMC13144603; doi:10.1038/s41377-026-02280-y)
Supplement: Supplementary file 1 — Supplementary information for Active steering of cathodoluminescence through a generalized Smith–Purcell effect [file 41377_2026_2280_MOESM1_ESM.pdf]

Active steering of cathodoluminescence through a generalized Smith–Purcell effect

– SUPPLEMENTARY INFORMATION –

Eduardo J. C. Dias [1,*] A. Rodríguez Echarri [2,3] Theis P. Rasmussen [1]
F. Javier García de Abajo [4,5] and Joel D. Cox [1,6,†]

[1] *POLIMA—Center for Polariton-driven Light–Matter Interactions, University of Southern Denmark, Campusvej 55, DK-5230 Odense M, Denmark*
[2] *Center for Nanophotonics, NWO Institute AMOLF, 1098 XG Amsterdam, The Netherlands*
[3] *Max-Born-Institut, 12489 Berlin, Germany*
[4] *ICFO-Institut de Ciencies Fotoniques, The Barcelona Institute of Science and Technology, 08860 Castelldefels (Barcelona), Spain*
[5] *ICREA-Institució Catalana de Recerca i Estudis Avançats, Passeig Lluís Companys 23, 08010 Barcelona, Spain*
[6] *Danish Institute for Advanced Study, University of Southern Denmark, Campusvej 55, DK-5230 Odense M, Denmark*

This Supplementary Information presents a full derivation of the dipole induced on a ribbon by a passing electron, the self-consistent line dipole–line dipole interaction formalism in translationally invariant systems, explicit expressions for the Green tensors and polarizability retrieval, and additional figures illustrating material-dependent responses and inverse-designed dipole profiles.

CONTENTS

S1. Electron interacting with an array of ribbons — S1
A. Induced dipole on a single ribbon — S1
B. Line dipole–line dipole interaction — S3

S2. Vanadium dioxide phase-changing dynamics — S4

S3. Supplementary figures — S5

References — S8

S1. ELECTRON INTERACTING WITH AN ARRAY OF RIBBONS

A. Induced dipole on a single ribbon

We follow the so-called plasmon wave function formalism [1–3], which applies to the specific case of a two-dimensional (2D) ribbon excited by an electron beam [4, 5]. We assume that the ribbon has a finite width W along the x direction, extends infinitely along the y-direction at the $z=0$ plane, and is described by a local optical conductivity $\sigma(\omega)$ at frequency ω. Within the quasistatic approximation, the electric field $\mathbf{E}(\mathbf{R},\omega)$ at the 2D ribbon coordinate $\mathbf{R}=(x,y)$ under illumination by a field $\mathbf{E}^{\rm ext}(\mathbf{R},\omega)$ can be written self-consistently as

$$\vec{\mathcal{E}}(\vec{\theta}) = \vec{\mathcal{E}}^{\rm ext}(\vec{\theta}) + \eta(\omega)\int {\rm d}^2\vec{\theta}\,\mathbf{M}(\vec{\theta},\vec{\theta}')\cdot\vec{\mathcal{E}}(\vec{\theta}'), \tag{S1}$$

where we define $\vec{\theta}=\mathbf{R}/W=(\theta_x,\theta_y)$, $\vec{\mathcal{E}}(\vec{\theta})=W\mathbf{E}(\vec{\theta},\omega)$, $\vec{\mathcal{E}}^{\rm ext}(\vec{\theta})=W\mathbf{E}^{\rm ext}(\vec{\theta},\omega)$, and $\mathbf{M}(\vec{\theta},\vec{\theta}')=(\nabla_{\vec{\theta}}\otimes\nabla_{\vec{\theta}})\,|\vec{\theta}-\vec{\theta}'|^{-1}$. Due to the translational invariance of the system along y, it is convenient to expand the fields as

$$\vec{\mathcal{E}}(\vec{\theta}) = \frac{1}{2\pi}\int {\rm d}Q\,\vec{\mathcal{E}}_Q(\theta_x){\rm e}^{{\rm i}Q\theta_y}, \tag{S2}$$

[*] Eduardo J. C. Dias: dias@mci.sdu.dk
[†] Joel D. Cox: cox@mci.sdu.dk

where each $Q = qW$ denotes a normal mode of the system with wave vector q along $\hat{\mathbf{y}}$. By doing so, we can rewrite Eq. (S1) as

$$\vec{\mathcal{E}}_Q(\theta_x) = \vec{\mathcal{E}}_Q^{\rm ext}(\theta_x) + \eta(\omega) \int_{-1/2}^{1/2} {\rm d}\theta_x \, \tilde{\mathbf{M}}_Q(\theta_x, \theta_x') \cdot \vec{\mathcal{E}}_Q(\theta_x'), \tag{S3}$$

where the operator $\tilde{\mathbf{M}}_Q(\theta_x, \theta_x') = 2(\nabla_{\vec{\theta}} \otimes \nabla_{\vec{\theta}}) K_0(|Q||\theta_x - \theta_x'|)$ is expressed in terms of the modified Bessel function K_0 [4], $\vec{\mathcal{E}}_Q^{\rm ext}(\theta_x) = \int {\rm d}\theta_y \, \vec{\mathcal{E}}^{\rm ext}(\vec{\theta}) {\rm e}^{-{\rm i}Q\theta_y}$.

In the absence of an external field, Eq. (S3) becomes an eigenvalue problem,

$$\vec{\mathcal{E}}_{mQ}(\theta_x) = \eta_{mQ} \int_{-1/2}^{1/2} {\rm d}\theta_x' \, \tilde{\mathbf{M}}_Q(\theta_x, \theta_x') \cdot \vec{\mathcal{E}}_{mQ}(\theta_x'), \tag{S4}$$

with eigenvalues η_{mQ} and associated eigenvectors $\vec{\mathcal{E}}_{mQ}(\theta_x)$ here presumed to be known (see discussion below). Using this relation, we can readily find that the Q-component of the self-consistent total electric field in Eq. (S3) within the ribbon can be written as

$$\vec{\mathcal{E}}_Q(\theta_x) = \sum_m \frac{c_{mQ}}{1 - \eta(\omega)/\eta_{mQ}}, \tag{S5}$$

with coefficients

$$c_{mQ} = \int_{-1/2}^{1/2} {\rm d}\theta_x \, \vec{\mathcal{E}}_Q^{\rm ext}(\theta_x) \cdot \vec{\mathcal{E}}_{mQ}(\theta_x). \tag{S6a}$$

We note that, in general, $\vec{\mathcal{E}}^{\rm ext}(\vec{\theta}) = -\nabla_{\vec{\theta}} \Phi^{\rm ext}(\vec{\theta})$ or, equivalently, $\vec{\mathcal{E}}_Q^{\rm ext}(\vec{\theta}) = -\nabla_{\theta_x} \Phi_Q^{\rm ext}(\theta_x)$, where we introduce $\nabla_{\theta_x} = (\partial_{\theta_x}, {\rm i}Q)$, with $\Phi_Q^{\rm ext}(\theta_x) = \int {\rm d}\theta_y \Phi^{\rm ext}(\vec{\theta}) {\rm e}^{-{\rm i}Q\theta_y}$ being the electrostatic potential associated with the external radiation. This allows us to evaluate the integral in Eq. (S6a) by parts to express it in the alternative form

$$c_{mQ} = \int_{-1/2}^{1/2} {\rm d}\theta_x \, \Phi_Q^{\rm ext}(\theta_x) \rho_{mQ}(\theta_x), \tag{S6b}$$

given in terms of the plasmon wave functions $\rho_{mQ}(\theta_x) = \nabla_{\theta_x} \cdot \vec{\mathcal{E}}_{mQ}(\theta_x)$.

Using the continuity equation ${\rm i}\omega\rho^{\rm ind}(\mathbf{R}) = \nabla \cdot \mathbf{J}(\mathbf{R})$ and Ohm's law $\mathbf{J}(\mathbf{R}) = \sigma(\omega)\mathbf{E}(\mathbf{R})$, the induced charge on the ribbon can be found as $\rho^{\rm ind}(\vec{\theta}) = (2\pi)^{-1} \int {\rm d}Q \, \rho_Q^{\rm ind}(\theta_x) {\rm e}^{{\rm i}Q\theta_y}$, where

$$\rho_Q^{\rm ind}(\theta_x) = \frac{1}{W} \sum_m \frac{c_{mQ}}{1/\eta_{mQ} - 1/\eta(\omega)} \rho_{mQ}(\theta_x). \tag{S7}$$

From here, the induced dipole moment density along the ribbon length can be written as $\mathbf{p}(\theta_y) = \int_{-1/2}^{1/2} {\rm d}\theta_x \, \rho^{\rm ind}(\vec{\theta})\vec{\theta}$. By using Eq. (S7) and employing a Fourier transform to bring the induced dipole moment density into the Q-space, $\mathbf{p}_Q = \int {\rm d}\theta_y \, \mathbf{p}(\theta_y) {\rm e}^{-{\rm i}Q\theta_y}$, we obtain the components

$$p_{Q,x} = W^2 \int_{-1/2}^{1/2} {\rm d}\theta_x \, \rho_Q^{\rm ind}(\theta_x)\theta_x = W \sum_m \frac{c_{mQ}}{1/\eta_{mQ} - 1/\eta(\omega)} \zeta_{mQ}, \tag{S8a}$$

$$p_{Q,y} = {\rm i}W^2 \partial_Q \int_{-1/2}^{1/2} {\rm d}\theta_x \, \rho_Q^{\rm ind}(\theta_x) = {\rm i}W \partial_Q \sum_m \frac{c_{mQ}}{1/\eta_{mQ} - 1/\eta(\omega)} \chi_{mQ}, \tag{S8b}$$

where we define $\zeta_{mQ} = \int_{-1/2}^{1/2} {\rm d}\theta_x \, \rho_{mQ}(\theta_x)\theta_x$ and $\chi_{mQ} = \int_{-1/2}^{1/2} {\rm d}\theta_x \, \rho_{mQ}(\theta_x)$, with the operator ∂_Q arising from an integration by parts. Importantly, χ_{mQ} vanishes when $Q = 0$ due to charge neutrality, but the same is not true when $Q \neq 0$.

The combination of Eqs. (S8) with Eq. (S6a) determines the dipole density along y induced on the ribbon by an incident electric field Q-component $\vec{\mathcal{E}}_Q^{\rm ext}(\theta_x)$. However, when the width W is much smaller than the wavelength $\lambda = 2\pi c/\omega$, we can neglect the variation of the incident field across the ribbon width and approximate $\vec{\mathcal{E}}_Q^{\rm ext}(\theta_x) \approx \vec{\mathcal{E}}_Q^{\rm ext}(0) \equiv \vec{\mathcal{E}}_Q^0 = (\mathcal{E}_{Q,x}^0, \mathcal{E}_{Q,y}^0)$. Under such conditions, we can write $\Phi_Q^{\rm ext}(\theta_x) \approx -[\theta_x \mathcal{E}_{Q,x}^0 + ({\rm i}Q)^{-1} \mathcal{E}_{Q,y}^0]$, from where we

obtain from Eq. (S6b) that $c_{mQ} = -\zeta_{mQ}\mathcal{E}^0_{Q,x} + (\mathrm{i}/Q)\chi_{mQ}\mathcal{E}^0_{Q,y}$. Plugging this result into Eqs. (S8) and noting that we can write, in general, $\mathbf{p}_Q = \overleftrightarrow{\alpha}_Q \cdot \vec{\mathcal{E}}^0_Q/W$, we can finally define the Q-component polarizability tensor $\overleftrightarrow{\alpha}_Q$ as

$$\overleftrightarrow{\alpha}_Q = W^2 \sum_m \begin{bmatrix} \zeta^2_{mQ} & \dfrac{1}{\mathrm{i}Q}\zeta_{mQ}\chi_{mQ} \\ \mathrm{i}\partial_Q \zeta_{mQ}\chi_{mQ} & \partial_Q \dfrac{1}{Q}\chi^2_{mQ} \end{bmatrix} \frac{1}{1/\eta(\omega) - 1/\eta_{mQ}}. \tag{S9}$$

We note that the ∂_Q operator acts on every Q-function to the right-hand side of it (including the $(1/\eta(\omega) - 1/\eta_{mQ})^{-1}$ part). For $Q = 0$, only the tensor component xx is nonzero and takes the form of Eq. (16) in the main text.

Using a Chebyshev polynomials basis described in Ref. 4, the eigenmodes of the system can be expanded as

$$\vec{\mathcal{E}}_{mQ}(\theta_x) = \sum_{n=0}^{\infty} \sqrt{1 - 4\theta_x^2}\, U_n(2\theta_x)(u_{mQ,n}, -\mathrm{i}v_{mQ,n}), \tag{S10}$$

where U_n denotes the Chebyshev polynomial of the second kind and $u_{mQ,n}$ and $v_{mQ,n}$ are expansion coefficients that can be determined as described in the mentioned reference, together with its associated eigenvalues η_{mQ}. In this basis, the plasmon wave functions are written as

$$\rho_{mQ}(\theta_x) = \sum_{n=0}^{\infty} \left[-2u_{mQ,n}(n+1)\frac{T_{n+1}(2\theta_x)}{\sqrt{1 - 4\theta_x^2}} + Qv_{mQ,n}\sqrt{1 - 4\theta_x^2}\, U_n(2\theta_x) \right], \tag{S11}$$

where T_n is a Chebyshev polynomial of the first kind. From the orthogonality conditions of these polynomials,

$$\int_{-1}^{1} dx\, \frac{T_n(x)T_m(x)}{\sqrt{1 - x^2}} = \frac{\pi}{2}\delta_{nm}(1 + \delta_{n0}), \tag{S12a}$$

$$\int_{-1}^{1} dx\, U_n(x)U_m(x)\sqrt{1 - x^2} = \frac{\pi}{2}\delta_{nm}, \tag{S12b}$$

and noting that $T_0(x) = U_0(x) = 1$ and $T_1(x) = U_1(x)/2 = x$, we can evaluate the integrals of $\rho_{mQ}(\theta_x)$ across the ribbon to derive

$$\zeta_{mQ} = -\frac{\pi}{4}u_{mQ,0} + Q\frac{\pi}{16}v_{mQ,1}, \tag{S13a}$$

$$\chi_{mQ} = Q\frac{\pi}{4}v_{mQ,0}. \tag{S13b}$$

As anticipated, χ_{mQ} vanishes for $Q = 0$, and we obtain $\zeta_{m0} = -(\pi/4)u_{mQ,0}$.

The coefficients $u_{mQ,n}$ and $v_{mQ,n}$, along with their associated eigenvalues η_{mQ}, can be found by the method described in Ref. 4, and for $Q = 0$ are tabulated in Tables S1 and S2 of its SI. For the dipolar mode in particular ($m = 1$), we have $\eta_{1,0} = -0.069$ and $u_{1,0,0} = -1.200$, yielding $\zeta_{1,0} = 0.942$. These parameters coincide with those reported in Ref. 2 for a ribbon illuminated by a plane wave, since the $Q = 0$ component of an electron beam field decomposition is equivalent to a normally-impinging plane wave.

B. Line dipole–line dipole interaction

We consider a line extending along $\hat{\mathbf{y}}$ and intersecting the point $\mathbf{r}'_\perp = (x', 0, z')$, carrying a dipole moment density $\mathbf{P}(y)$ per unit length along the y-direction. The field generated by such line at position $\mathbf{r} = (x, y, z)$ can be written as

$$\mathbf{E}^{\mathrm{dip}}(\mathbf{r}) = \int \mathrm{d}y'\, (k^2 + \nabla_\mathbf{r} \otimes \nabla_\mathbf{r})\frac{\mathrm{e}^{\mathrm{i}k|\mathbf{r}-\mathbf{r}'|}}{|\mathbf{r} - \mathbf{r}'|} \cdot \mathbf{P}(y'), \tag{S14}$$

with $\mathbf{r}' = \mathbf{r}'_\perp + y'\hat{\mathbf{y}}$, $\nabla_\mathbf{r} = (\partial_x, \partial_y, \partial_z)$, and we have used the point dipole–point dipole interaction Green's tensor (see main text). Now, similarly to the previous section, we employ a Fourier transform to write $\mathbf{P}(y) = (2\pi)^{-1}\int \mathrm{d}q\, \mathbf{P}_q \mathrm{e}^{\mathrm{i}qy}$. Replacing this in the equation above and using the identity

$$\int \mathrm{d}y' \frac{\mathrm{e}^{\mathrm{i}k|\mathbf{r}-\mathbf{r}'|}}{|\mathbf{r} - \mathbf{r}'|}\mathrm{e}^{\mathrm{i}qy'} = \frac{\mathrm{i}}{4}H_0^{(1)}(\kappa|\mathbf{r}_\perp - \mathbf{r}'_\perp|)\mathrm{e}^{\mathrm{i}qy}, \tag{S15}$$

expressed in terms of the Hankel function $H_0^{(1)}$, with $\kappa = \sqrt{k^2 - q^2}$ and $\mathbf{r}_\perp = (x, 0, z)$, we readily find that $\mathbf{E}_q^{\rm ind}(\mathbf{r}_\perp) = \int {\rm d}y\, \mathbf{E}^{\rm ind}(\mathbf{r}) {\rm e}^{-{\rm i}qy} = \mathcal{G}_q(\mathbf{r}_\perp, \mathbf{r}'_\perp) \cdot \mathbf{P}_q$, such that the line dipole–line dipole interaction Green's tensor associated with wave vector component q can be written as

$$\mathcal{G}_q(\mathbf{r}_\perp, \mathbf{r}'_\perp) = \frac{\rm i}{4}(k^2 + \nabla_\perp \otimes \nabla_\perp) H_0^{(1)}(\kappa|\mathbf{r}_\perp - \mathbf{r}'_\perp|), \tag{S16}$$

where $\nabla_\perp = (\partial_x, {\rm i}q, \partial_z)$. By explicitly performing the derivations, the Green tensor can be expressed in the closed form

$$\mathcal{G}_q(\mathbf{r}_\perp, \mathbf{r}'_\perp) = \frac{{\rm i}\kappa^2}{4}\left[C_\rho(\hat{\rho} \otimes \hat{\rho}) + C_\phi(\hat{\phi} \otimes \hat{\phi}) + \frac{k^2}{\kappa^2} H_0(\hat{\mathbf{y}} \otimes \hat{\mathbf{y}}) - {\rm i}\frac{q}{\kappa} H_1(\hat{\rho} \otimes \hat{\mathbf{y}} + \hat{\mathbf{y}} \otimes \hat{\rho})\right], \tag{S17}$$

where $\vec{\rho} = \mathbf{r}_\perp - \mathbf{r}'_\perp$, $\hat{\rho} = \vec{\rho}/\rho$, $\rho = |\vec{\rho}|$, $\hat{\phi} = \hat{\mathbf{y}} \times \hat{\rho}$, $H_\nu = H_\nu^{(1)}(\kappa\rho)$, and we introduce

$$C_\rho = \left(\frac{k^2}{\kappa^2} + \frac{3}{2}\right) H_0 - \frac{1}{2} H_2, \tag{S18}$$

$$C_\phi = \left(\frac{k^2}{\kappa^2} - \frac{1}{2}\right) H_0 + \frac{1}{2} H_2. \tag{S19}$$

For the results presented in the main text, we are primarily interested in the xx component of the tensor for $q = 0$ and for $z = z' = 0$, for which we find

$$\mathcal{G}_0^{xx}(x, x') = \frac{{\rm i}k^2}{2}\left[H_0^{(1)}(k|x - x'|) - \frac{1}{2k|x - x'|} H_1^{(1)}(k|x - x'|)\right]. \tag{S20}$$

S2. VANADIUM DIOXIDE PHASE-CHANGING DYNAMICS

The insulator–metal transition (IMT) in VO_2 is triggered by an increase in temperature T, with the metallic fraction $f_{\rm m}$ being well described by a sigmoidal law

$$f_{\rm m}(T) = \frac{1}{1 + \exp[-(T - T_{\rm m})/\Delta T]}, \tag{S21}$$

where $T_{\rm m} \approx 341$ K and $\Delta T \approx 2$ K [6] (see Fig. S5(c)). This dependence reflects the dynamics of the phase-change: little variation below the IMT window, followed by a rapid rise once nucleation, growth, and percolation of metallic domains activate, and saturation as the remaining insulating volume is exhausted [6–8]. As the temperature decreases, the material relaxes back toward the insulating state. Because the IMT in VO_2 is a first-order phase transition, it exhibits thermal hysteresis [8–10], so the parameters stated above for Eq. (S21) do not describe the cooling branch (which is also sigmoidal, but with its own onset, midpoint, and width). However, keeping hysteresis into account, complete tuning can be achieved with an appropriate heating/cooling protocol.

To estimate the temperature reached by a small VO_2 sample with volume V and absorption cross section $\sigma_{\rm abs}(\omega_{\rm p})$ under pumping by a short light pulse with frequency $\omega_{\rm p}$ and fluence F_0, we solve the adiabatic energy-balance [11]

$$F_0 = V \int_{T_0}^{T} dT' \frac{c_V}{\sigma_{\rm abs}(\omega_{\rm p})}, \tag{S22}$$

with T_0 being the ambient temperature, and $c_V \approx 3.0\ {\rm J\,cm^{-3}\,K^{-1}}$ [12] the heat capacity per unit volume of VO_2 (which we take as approximately independent of T for the purpose of this estimation). We note that $\sigma_{\rm abs}$ depends on temperature via the variation in permittivity of the sample at the pump wavelength as the IMT occurs. Applying this expression to VO_2 disk with the characteristics specified in the main text, we obtain a fluence-dependent $f_{\rm m}$ that can be accurately described by with a sigmoidal function with the form shown in Fig. S5(d), described by Eq. (19) in the main text (with the parameters therein indicated).

For higher accuracy, the simple temperature estimate above can be replaced by a time-resolved thermal model (eg. the two-temperature model [13–15]), including an accurate temperature-dependence of the heat capacity $c_V(T)$. The resulting $T(t)$ curve can then be used with first-order IMT kinetics, namely the Johnson-Mehl-Avrami-Kolmogorov (JMAK) growth model [13, 14] in combination with an Arrhenius temperature dependence [14, 16], to determine the final metallic fraction for a given pump pulse characteristics.

S3. SUPPLEMENTARY FIGURES

We include in this section the following supplementary figures:

- **Figs. S1 and S2**: counterparts of Figs. 2 and 3 in the main text, respectively, for arrays with $N = 101$ elements (instead of 51).
- **Figs. S3 and S4:** additional properties of the engineered GSP radiation.
- **Fig. S5:** thermo-optical properties of VO_2.
- **Fig. S6:** polarization tuning of the different array elements considered in Figs. S7 and 4 in the main text.
- **Fig. S7:** counterpart of Fig. 4 in the main text, but showcasing passive array engineering using silver nanorods and gold ribbons.

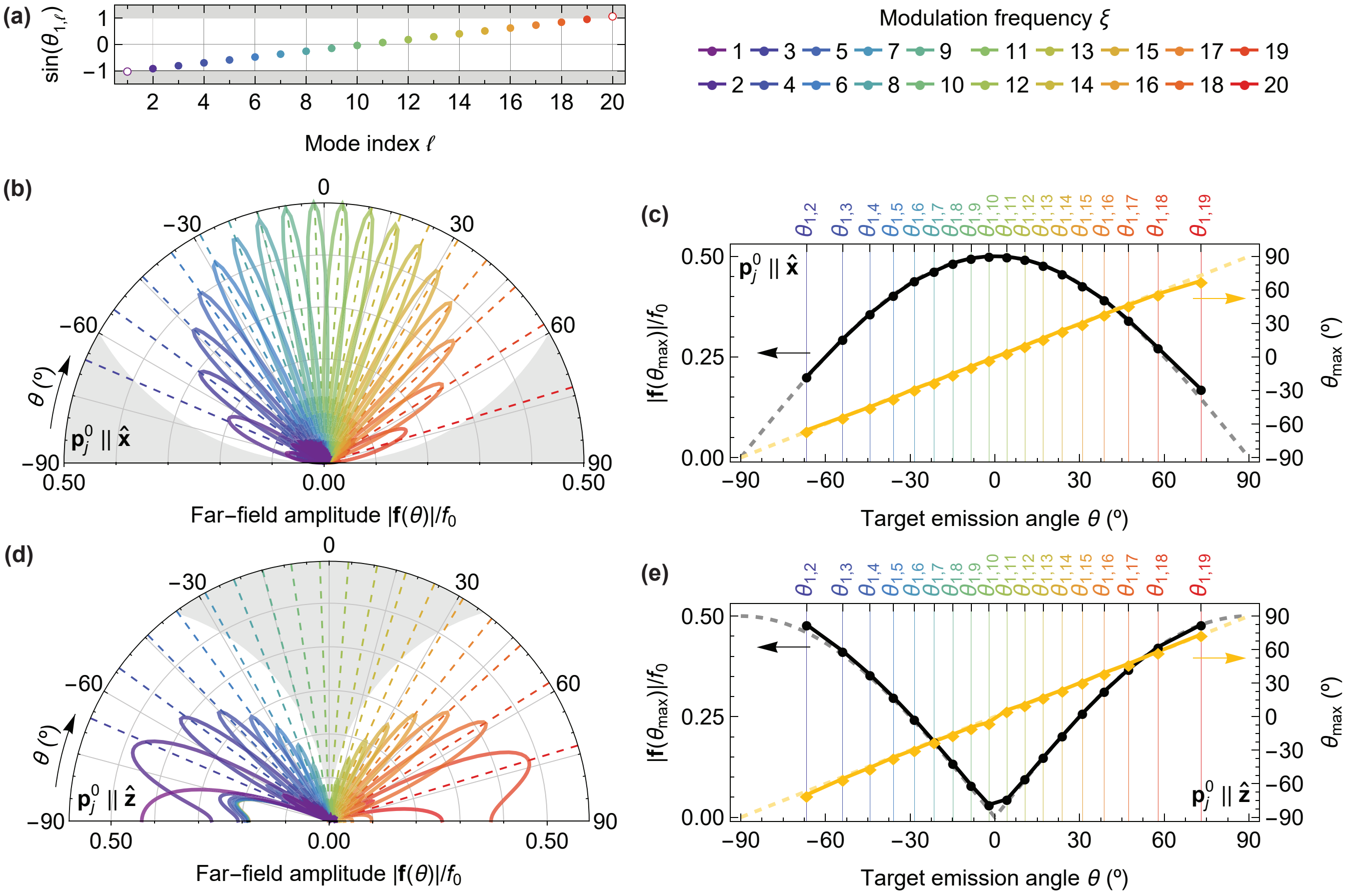

FIG. S1. **Cathodoluminescence steering with large non-uniform arrays.** **(a)** GSP condition in Eq. (6) in the main text for $n = 1$ and $\ell = 1 - 20$ in an array with $N = 101$ elements and period $a/\lambda = 0.090$, excited by an electron beam with velocity $v = 0.1c$ (≈ 2.6 keV). Modes $\ell = 2 - 19$ lie within the region $|\sin\theta_{1,\ell}| \leq 1$ and are marked by filled circles, whereas modes $\ell = 1$ and $\ell = 20$ lie outside the same region and are marked with empty circles. **(b)** Angle-resolved cathodoluminescence far-field emission amplitude $|\mathbf{f}(\theta)|$ polar plot, normalized to $f_0 = Nk^2p_0$, as a function of θ angle in the $\phi = 0$ plane, for an electron passing over an array whose induced dipole moments are given by Eq. (8) in the main text with ξ as indicated in the legend and polarized along x ($\mathbf{p}_j^0 \parallel \hat{\mathbf{x}}$). The colored dashed lines mark the position of the target angles $\theta_{1,\xi}$ for $\xi = 2 - 19$ and the shaded gray area represents the condition $|\mathbf{f}(\theta)|/f_0 > \cos\theta$. **(c)** Peak emission angle $\theta_{\rm max}$ (right axis) and corresponding peak far-field amplitude $|\mathbf{f}(\theta_{\rm max})|/f_0$ (left axis) as a function of the target emission frequency θ for $\xi = 2 - 19$ (see top axis). The dashed gray curve represents the function $|\mathbf{f}(\theta)|/f_0 = \cos(\theta)/2$ and the yellow dashed line represents the condition $\theta_{\rm max} = \theta$. **(d,e)** Same as (b,c), respectively, but for dipoles polarized along z ($\mathbf{p}_j^0 \parallel \hat{\mathbf{z}}$). In (d), the shaded gray area represents the condition $|\mathbf{f}(\theta)|/f_0 > \sin\theta$. In (e), the dashed gray curve represents the function $|\mathbf{f}(\theta)|/f_0 = \sin(\theta)/2$.

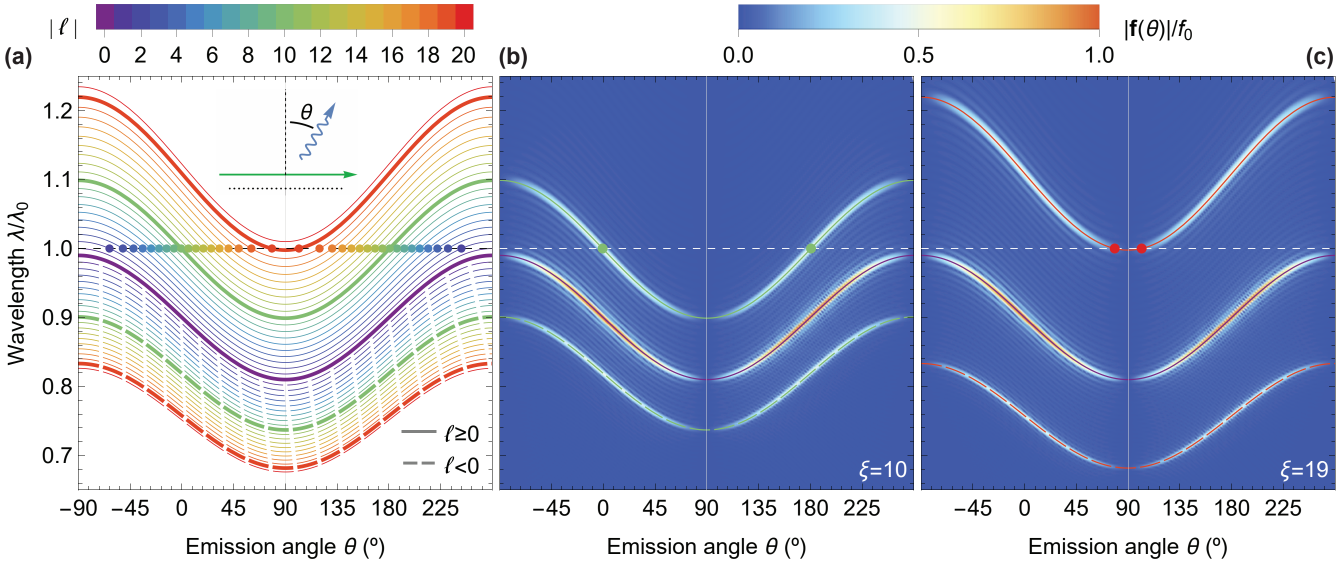

FIG. S2. **Wavelength dependence of GSP emission in large non-uniform arrays.** **(a)** Relation between wavelength λ and emission angle θ for GSP modes labeled by $-20 \leq \ell \leq 20$ (see legends) and $n = 1$. The electron velocity is $v = 0.1c$, and we fix $a/\lambda_0 = 0.09$ and $N = 101$. The curves corresponding to $\ell = 0, \pm 10, \pm 19$ are highlighted to facilitate the comparison with panels (b) and (c). The black dashed line corresponds to $\lambda = \lambda_0$ (i.e., the same condition as in Fig. S1), and the dots mark its intersection with each color-coordinated curve. The inset shows angle θ of light emission (blue arrow) relative to the array (black dots) and electron trajectory (green arrow). **(b,c)** Far-field emission intensity $|\mathbf{f}(\theta)|$, as a function of θ and λ and normalized to $f_0 = Nk^2p_0$, for an array whose dipole moment distribution follows Eq. (8) in the main text with (b) $\xi = 10$ and (c) $\xi = 19$. In each case, we faintly overlay the same curves and circular dots as in panel (a) corresponding to $\ell = 0, \pm\xi$.

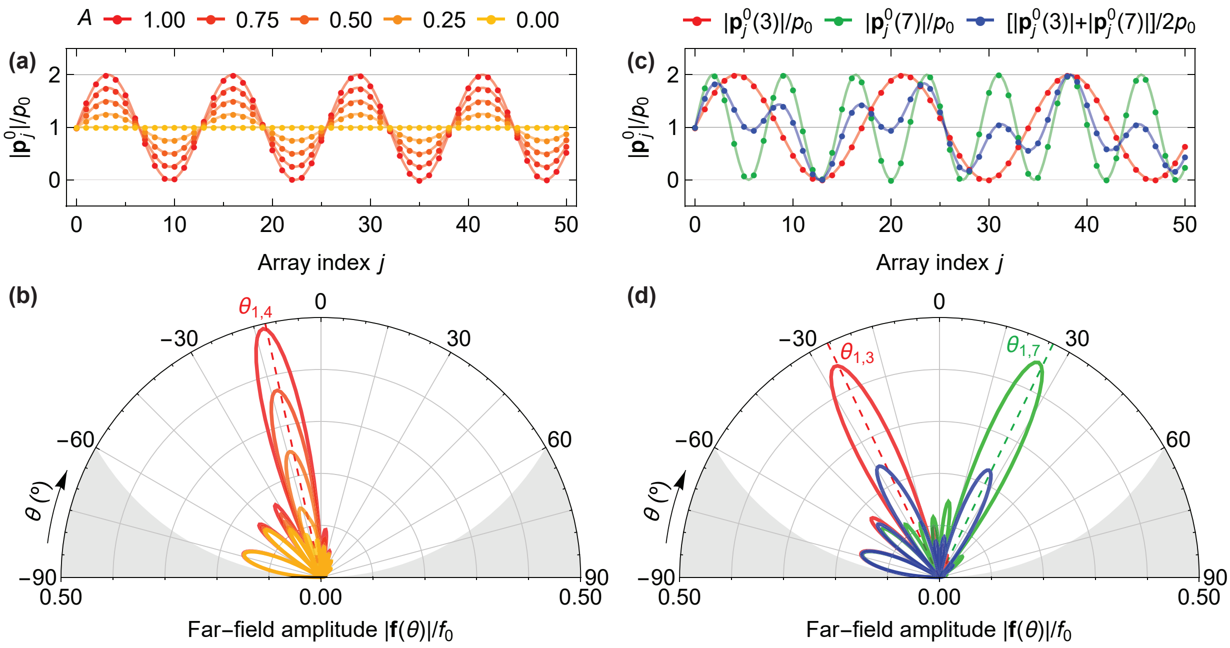

FIG. S3. **Properties of the CL far-field emission profile.** **(a)** Induced dipole distribution profile following Eq. (8) in the main text with $\xi = 4$, with the parameter A ranging from 0 to 1 (see legend). **(b)** CL angle-resolved far-field distribution $|\mathbf{f}(\theta)|$ at $\phi = 0$ for each of the color-coded distributions in (a). **(c,d)** Same as (a,b), respectively, but for induced dipole profiles following Eq. (8) with $A = 1$ for $\xi = 3$, $\xi = 7$, and the averaged superposition of both profiles, as shown in the legend.

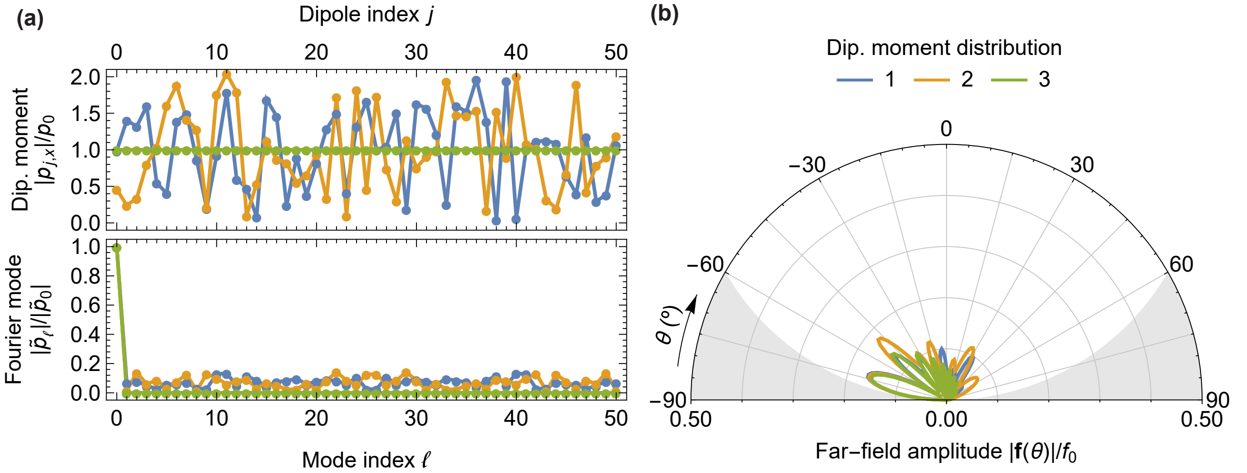

FIG. S4. **Random dipole distributions.** **(a)** Dipole moment distributions (top) and corresponding Fourier decomposition (bottom) in arrays with $N = 51$ dipoles constructed randomly (blue and orange, labeled 1 and 2) or uniformly (green, labeled 3). All three distributions exhibit the same total dipole moment Np_0. **(b)** Angle-resolved far-field CL distribution $|\mathbf{f}(\theta)|$ at $\phi = 0$ for each of the color-coded distributions in panel (a), with parameters $a/\lambda_0 = 0.09$ and $v = 0.1c$, normalized to $f_0 = Nk^2p_0$. In panel (b), we maintain the same scale range as in Figs. 2, 4, S1, S3, and S7 in the radial direction to help the comparison of the CL far-field amplitude between all the figures.

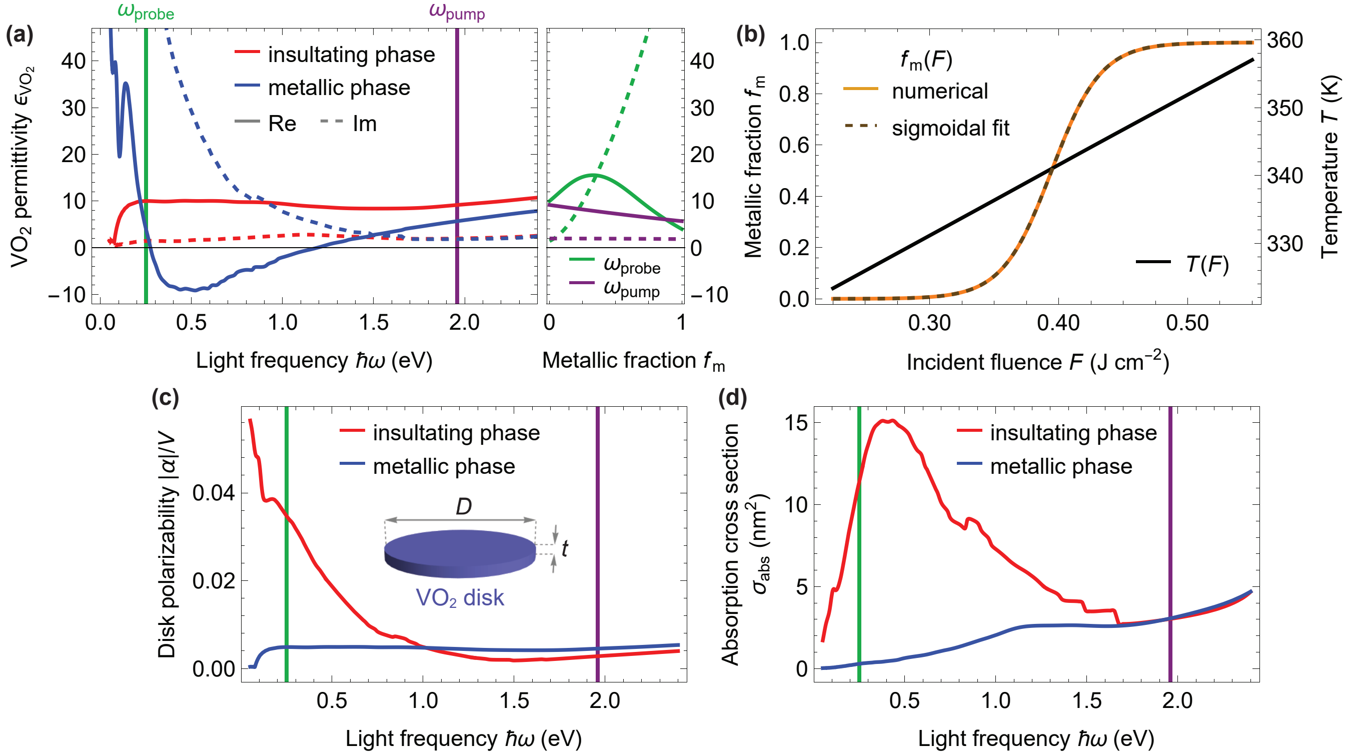

FIG. S5. **VO_2 thermo-optical properties.** **(a)** Real and imaginary parts of the permittivity of VO_2 in its insulating ($f_{\rm m} = 0$) and metallic ($f_{\rm m} = 1$) phases. On the right panel, we show the variation of the permittivity with $f_{\rm m}$ for two representative frequencies $\hbar\omega_{\rm probe} = 0.25$ eV and $\hbar\omega_{\rm pump} = 1.96$ eV, marked by vertical lines on the left plot. **(b)** Variation of the temperature (right axis) and corresponding metallic fraction $f_{\rm m}$ (left axis) of a VO_2 disk with $D = 250$ nm and $t = 2$ nm (see inset in (c)) as a function of incident fluence F at $\omega_{\rm pump}$. **(d)** Disk polarizability $|\alpha|$ (normalized to its volume $V = \pi D^2 t/4$) and **(e)** absorption cross section $\sigma_{\rm abs}$, as a function of light frequency, for the insulating and metallic phases.

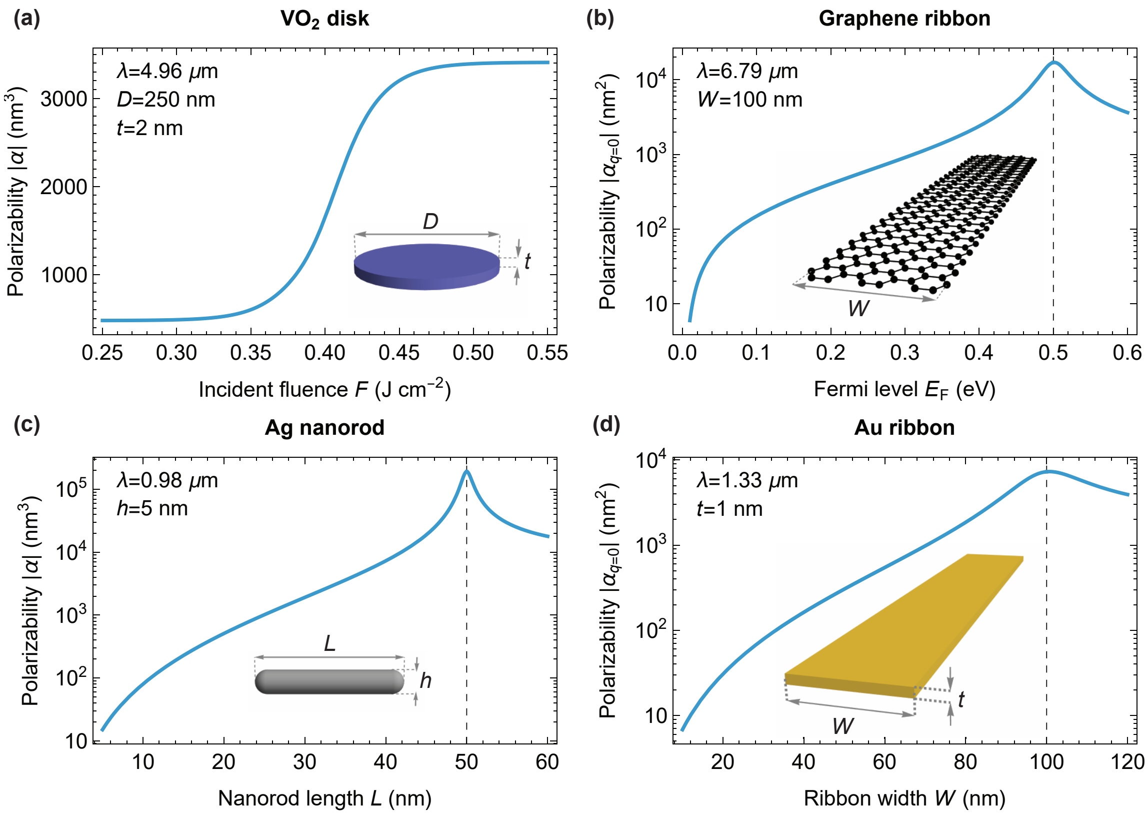

FIG. S6. **Polarizability tuning.** **(a)** Variation of the polarizability amplitude $|\alpha|$ of a VO_2 disk with diameter D and thickness t (see scheme and labels), as a function of incident fluence F for pumping at 632 nm. **(b)** Variation of the effective polarizability at $q = 0$ (see Methods and SI) of a graphene ribbon with width W, as a function of Fermi energy E_F. **(c,d)** Same as (a,b), respectively, but for (c) a silver nanorod of length L and tip diameter h, as a function of L, and (d) a gold ribbon with thickness t and width W as a function of W. In all plots, the wavelength at which the polarizability is calculated is indicated by a label.

REFERENCES

[1] F. J. García de Abajo, "Multiple excitation of confined graphene plasmons by single free electrons," ACS Nano **7**, 11409–11419 (2013).

[2] I. Silveiro, J. M. Plaza Ortega, and F. J. García de Abajo, "Plasmon wave function of graphene nanoribbons," New J. Phys. **17**, 083013 (2015).

[3] R. Yu, J. D. Cox, J. R. M. Saavedra, and F. J. García de Abajo, "Analytical modeling of graphene plasmons," ACS Photonics **4**, 3106–3114 (2017).

[4] V. Mkhitaryan, E. J. C. Dias, F. Carbone, and F. J. García de Abajo, "Ultrafast momentum-resolved free-electron probing of optically pumped plasmon thermal dynamics," ACS Photonics **8**, 614–624 (2021).

[5] T. P. Rasmussen, A. Rodríguez Echarri, J. D. Cox, and F. J. García de Abajo, "Generation of entangled waveguided photon pairs by free electrons," Sci. Adv. **10**, eadn6312 (2024).

[6] Peter Uhd Jepsen, Bernd M. Fischer, Andreas Thoman, Hanspeter Helm, J. Y. Suh, René Lopez, and Jr. Haglund, R. F., "Metal-insulator phase transition in a VO_2 thin film observed with terahertz spectroscopy," Physical Review B **74**, 205103 (2006).

[7] M. M. Qazilbash, M. Brehm, Byung-Gyu Chae, P.-C. Ho, G. O. Andreev, Bong-Jun Kim, Sun Jin Yun, A. V. Balatsky, M. B. Maple, F. Keilmann, Hyun-Tak Kim, and D. N. Basov, "Mott transition in VO_2 revealed by infrared spectroscopy and nano-imaging," Science **318**, 1750–1753 (2007).

[8] H. W. Verleur, Jr. Barker, A. S., and C. N. Berglund, "Optical properties of VO_2 between 0.25 and 5 eV," Physical Review **172**, 788–798 (1968).

[9] C. L. Gomez-Heredia, J. A. Ramirez-Rincon, J. Ordonez-Miranda, O. Ares, J. J. Alvarado-Gil, C. Champeaux, F. Dumas-Bouchiat, Y. Ezzahri, and K. Joulain, "Thermal hysteresis measurement of the VO_2 emissivity and its application in thermal rectification," Scientific Reports **8**, 8479 (2018).

[10] Georges Hamaoui, Nicolas Horny, Cindy Lorena Gomez-Heredia, J. A. Ramirez-Rincon, Mihai Chirtoc, Younès Ezzahri, and Karl Joulain, "Thermophysical characterisation of VO_2 thin films: hysteresis and its application in thermal rectification," Scientific Reports **9**, 8728 (2019).

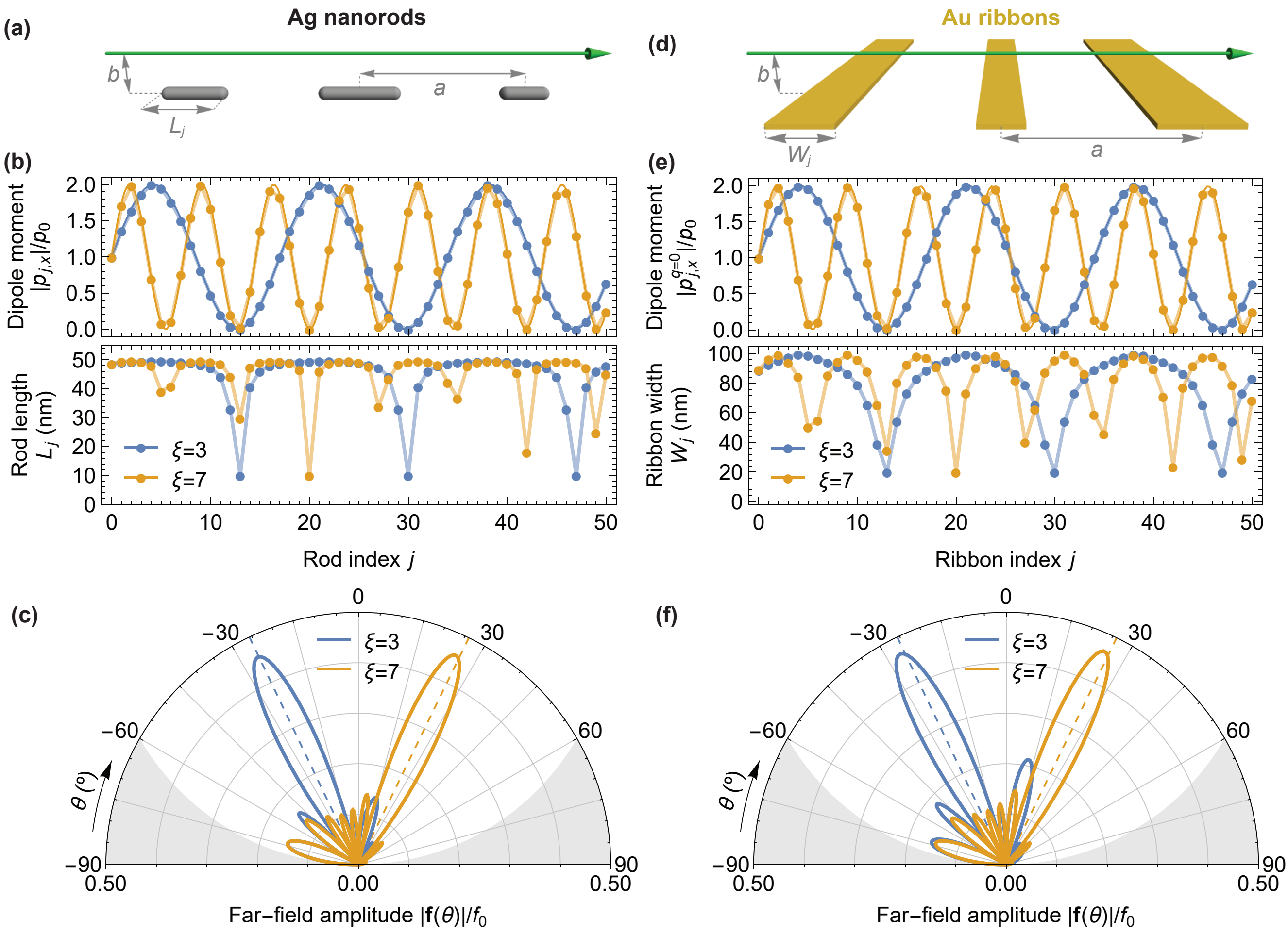

FIG. S7. **Passive tuning of CL emission.** **(a)** Scheme of an array of silver nanorods with thickness $h = 5$ nm and length L_j, separated from their nearest neighbors by a center-to-center distance $a = 90$ nm, with an electron passing parallel to the array at a distance $b = 10$ nm and with velocity $v = 0.1c$. **(b)** Induced dipole moment (top) at wavelength $\lambda = 2\pi c/\omega = 985$ nm (≈ 1.26 eV) on the array, with $p_0 = 9.45(eL_{\max}/\omega)$, as a function of element j, for the color-coordinated rod length distribution L_j (bottom) ranging from $L_{\min} = 10$ nm to $L_{\max} = 50$ nm [17]. **(c)** Far-field emission distribution for the same-color array distributions in (b), with $f_0 = 50.0(e/L_{\max}\omega)$. **(d-f)** Same as (a-c), but for an array of gold ribbons with thickness $t = 1$ nm and width W_j ranging from $W_{\min} = 20$ nm to $W_{\max} = 100$ nm [18], with $a = 120$ nm and $\lambda = 1.33$ μm (≈ 0.93 eV). In (e), where the dipole moment corresponding to $q = 0$ component, with $p_0 = 6.72(eW_{\max}/\omega)$. In (f), we have $f_0 = 76.1/(e/W_{\max}\omega)$.

[11] V. R. Voller and M. Cross, "Accurate solutions of moving boundary problems using the enthalpy method," International Journal of Heat and Mass Transfer **24**, 545–556 (1981).
[12] Dong-Wook Oh, Changhyun Ko, Shriram Ramanathan, and David G. Cahill, "Thermal conductivity and dynamic heat capacity across the metal-insulator transition in thin film VO_2," Applied Physics Letters **96**, 151906 (2010).
[13] Melvin Avrami, "Kinetics of phase change. I. General theory," The Journal of Chemical Physics **7**, 1103–1112 (1939).
[14] Jordi Farjas and Pere Roura, "Modification of the Kolmogorov–Johnson–Mehl–Avrami rate equation for non-isothermal experiments and its analytical solution," Acta Materialia **54**, 5573–5579 (2006).
[15] A. Pashkin, C. Kübler, H. Ehrke, R. Lopez, A. Halabica, Jr. Haglund, R. F., R. Huber, and A. Leitenstorfer, "Ultrafast insulator-metal phase transition in VO_2 studied by multiterahertz spectroscopy," Physical Review B **83**, 195120 (2011).
[16] Yimin Chen, Nan Han, Fanshuo Kong, Jun-Qiang Wang, Chenjie Gu, Yixiao Gao, Guoxiang Wang, and Xiang Shen, "Kinetics features of 2D confined $Ge_2Sb_2Te_5$ ultrathin film," Applied Physics Letters **121**, 061904 (2022).
[17] Amit Kumar, Muhammad Omar Shaikh, and Cheng-Hsin Chuang, "Silver nanowire synthesis and strategies for fabricating transparent conducting electrodes," Nanomaterials **11**, 693 (2021).
[18] Chenxinyu Pan, Yuanbiao Tong, Haoliang Qian, Alexey V Krasavin, Jialin Li, Jiajie Zhu, Yiyun Zhang, Bowen Cui, Zhiyong Li, Chenming Wu, *et al.*, "Large area single crystal gold of single nanometer thickness for nanophotonics," Nature Communications **15**, 2840 (2024).
